# Cancer-associated adipocytes as immunomodulators in cancer

**DOI:** 10.1186/s40364-020-00257-6

**Published:** 2021-01-07

**Authors:** Qi Wu, Bei Li, Juanjuan Li, Si Sun, Jingping Yuan, Shengrong Sun

**Affiliations:** 1 Department of Breast and Thyroid Surgery, Renmin Hospital of Wuhan University, 238 Ziyang Road, Wuhan, 430060 Hubei Province P. R. China; 2 Department of Pathology, Renmin Hospital of Wuhan University, 238 Ziyang Road, Wuhan, 430060 Hubei Province P. R. China; 3 Department of Clinical Laboratory, Renmin Hospital of Wuhan University, Wuhan, Hubei P. R. China

**Keywords:** Cancer-associated adipocytes, Tumor, Immune cells, Metabolism

## Abstract

Cancer-associated adipocytes (CAAs), as a main component of the tumor-adipose microenvironment (TAME), have various functions, including remodeling the extracellular matrix and interacting with tumor cells or infiltrated leukocytes through a variety of mutual signals. Here, we summarize the primary interplay among CAAs, the immune response and cancer with a focus on the mechanistic aspects of these relationships. Finally, unifying our understanding of CAAs with the immune cell function may be an effective method to enhance the efficacy of immunotherapeutic and conventional treatments.

## Introduction

Obesity is now considered to be the most crucial modifiable reason for cancer. Overweight and obesity (body mass index (BMI) 25.0–29.9, or ≥30 kg/m^2^, respectively) are correlated to elevated risk of colorectal cancer, postmenopausal breast cancer, and cancers of the endometrium, gall bladder, pancreas, kidney, and liver [1, 2]. However, not all cancers are related to obesity; higher BMI is correlated to lower risk of breast cancer instead of higher risk in premenopausal women [3]. The molecular mechanisms underlying risk stratification have not been well demonstrated and have motivated further molecular and cellular research on the functional associations between obesity and cancer.

Obesity exhibits excess accumulation of adipose tissue and results in dysfunctional adipose tissue. Some research has demonstrated that adipocytes, as the main element of the stromal microenvironment of multiple cancers, display tumor-promoting impacts on various tumor cells on a molecular level. In particular, cancer-associated adipocytes (CAAs) are thought to be essential factors in cancer progression, since they directly or indirectly facilitate cell growth, angiogenesis, anti-apoptotic effects and migration [4, 5]. CAAs are responsible mainly for metabolic storage. Lipids are deposited here as triacylglycerols (TAGs) and released in the form of free fatty acids (FFAs) when needed. In addition to energy storage, CAAs contribute to endocrine actively signaling to tumors by secreting hormones, cytokines, adipokines and growth factors [5–7]. Surprisingly, CAAs may profoundly affect the effector functions of immune cells. In the obese state, adipocytes become hypertrophic with increased storage of TAGs, and the excretion of adipokines and proinflammatory cytokines is also elevated, including IL-6, IL-8, tumor necrosis factor-α (TNF-α) and PAI-1. Monocytes, macrophages, and other immune cells are attracted by these molecules, thus stimulating the formation of chronic low-grade inflammation in the adipose tissue. Consequently, lipolysis starts, and adipocytes release more FFAs, which is not conducive to the lipid homeostasis of the entire organism and results in subsequent immune alterations [8]. Moreover, according to the nutrient and growth factors in the TME, many of these intracellular metabolic pathways are interchangeable, whereas other pathways are stringently necessary for a specific cell lineage, for example, in T regulatory cells (Tregs). The interaction between the immune system and adipocytes has been addressed by the identification of specific immune cell populations residing in adipose tissue and the metabolic or inflammatory factors secreted by these immune cells. This communication affects the local homeostasis profoundly, which subsequently plays a role in the differentiation and function of various immune cells.

In this review, we summarize the hallmarks of CAAs thus far. Moreover, we concentrate on the effect of CAAs on the resident immune cells and illustrate how the adipocyte-immune cell interplay drives tumor growth and progression.

### CAAs as a complex assembler

**Fig. 1:**
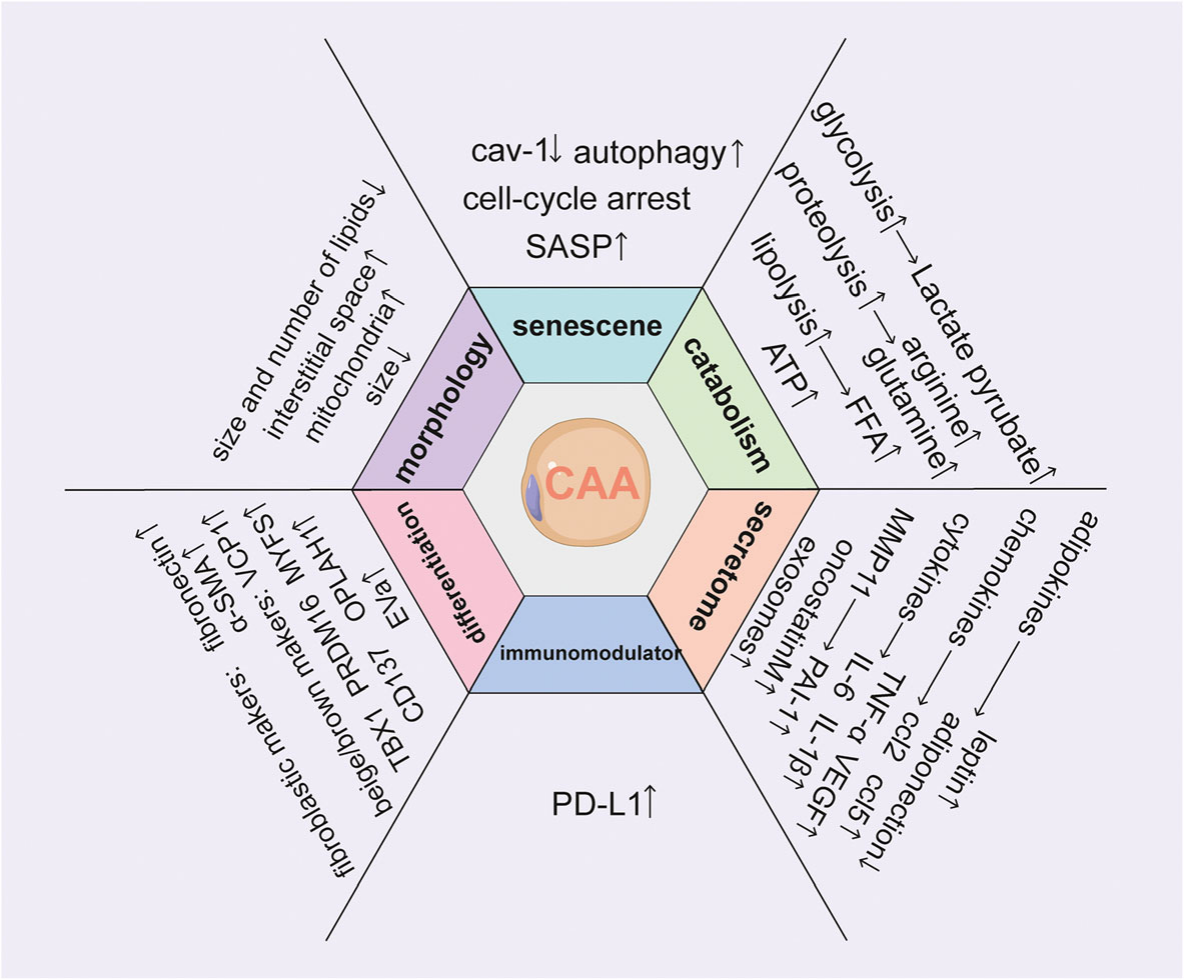
Typical characteristics of cancer-associated adipocytes (CAAs)

Compared to normal adipocytes, CAAs are characterized by a decrease in size and lipid content and adipocyte differentiation markers, as well as an increase of adipokines and inflammatory factors, such as leptin, matrix metalloproteinase (MMP)-11, CCL2, CCL5, IL-6 [9, 10]. CAAs have been traditionally considered the peritumoral adipocytes that display a modified phenotype and particular biological characteristics sufficient to support tumor progression [5, 9]. However, CAAs are now recognized to be a complex and dynamic process. Nonetheless, CAAs display their heterogeneity and plasticity, which, in aggregate, defines the CAA phenotypes (Fig. 1). Salient features of CAAs are:

**Table 1:** Important immune cells involved in CAA regulation

Immune cells	Mechanism	Alternations	References
Tumor-associated neutrophils (TANs)	Glycolysis↑ FFA uptake↑ A_3_R activation	↑differentiation ↑ARG1	[54, 56, 59]
Natural killer cells	MYC ↓ mTORC1↓→glycolysis↓, OXPHOS↓ Lipid accumulation↑	↓IFN-γ ↓Granzymes ↓Perforin ↑Apoptosis	[72, 73, 75, 77, 79, 83, 86]
Natural killer T cells	CD1d↓	↓Effector function	[92, 93]
Tumor-associated macrophages (TAMs)	HIF1α stabilization → glycolysis PPAR-γ, PGC-1β↑→FFA uptake, oxidation↑ GPR132 activation CD39 CD73↑→A_2_BR activation	↑M2-like polarization ↑ARG1 ↑VEGF	[109, 111, 115, 117]
Myeloid-derived suppressor cells (MDSCs)	PUFAs→ immune suppression↑ CSF→ lipid metabolism↑	↓T cell activation	[121, 122]
Dendritic cells	mTORC1/HIF1/NOS2↓→glycolysis↓ Lipid accumulation↑ PKA/Epac↑ GPR8 activation	↓antigen-presentation function ↑IL-10 ↓IL-12	[135, 137, 140, 142]
Regulatory T cells	CD36↑→ FFA uptake, oxidation↑ PPAR-γ↑ MCT1↑ → OXPHOS↑ CD39↑→A_2_AR activation	↑Differentiation ↑Proliferation	[165, 168, 172]
Effector T cells	Glycolysis↓ OXPHOS↓ CPT1α↑ → FAO↑	↓Effector function ↓Proliferation ↓Cytokine production ↑PD1	[173, 184, 185, 190, 193]

- Morphologically, CAAs exhibit small sizes with an extended interstitial space [9], which may be associated with extensive extracellular matrix in adipocytes surrounding tumor cells, such as the overexpression of collagen VI [11]. In addition, the size and number of intracellular lipid droplets are found to decrease observably [12]. More complex ultra-structures in the CAAs that can be seen with electron microscopy include significantly abundant mitochondria, which increases matrix density and expansion of the cristal spaces [13].
- CAAs possess senescent features. Recently, several studies have demonstrated that adipocytes can be diverted into a dysfunctional, proinflammatory, senescent-like phenotype in obese individuals [4, 14, 15]. Phenomenally, the main feature of CAAs is their cell-cycle arrest, alongside with the upregulation of genes associated with arrest cell cycle but the downregulation of genes known to induce proliferation [16]. Regarding the effects of oncogenic pathway activation on cell-cycle arrest in CAAs [17], it shows a remarkedly suppressed oncogenic pathways like cell cycle regulation, Myc transcription, and tyrosine receptor kinases signaling, but an increase expression of tumor-suppressive pathways like Hippo pathway in CAAs [16]. In addition to senescence-associated secretory phenotype (SASP), bone marrow adipose tissue (BMAT) in myeloma-burdened mice exhibits an increased senescent cytokines like IL-6, IL-8, CXCL1, and CXCL2 [18]. Mechanically, autophagy might have an essential effect on the malignant conversion and aging of stromal cells. For example, caveolin-1 (Cav-1) is the major structural protein for caveolae and functions as a vital factor in membrane transport (endocytosis and transcytosis), as well as the preservation of membrane lipid composition and signal transduction within cells [19]. Interestingly, Cav-1 is thought to be a tumor suppressor, and tumor growth and metastasis were facilitated by Cav-1 downregulation in adipocyte surrounded breast cancer cells [12, 20]. Under hypoxic conditions, autophagic response can degrade Cav-1 through stimulation of NF-κB and HIF-1a [21]. Likewise, low expression of Cav-1 is shown to result in the stimulation of cellular senescence of fibroblasts; the loss of Cav-1 diminished mitochondrial respiration and prevented silent information regulator 2 homolog 1 (SIRT1) from working, thus facilitating premature aging [22]. For that reason, autophagy induced by Cav-1 might be the essential crosstalk between CAAs and cellular senescence. Additionally, the transformation from normal adipocytes to CAAs may undergo cellular aging resulting from the activation of many oncogenes. Evidently, the injection of tumor cells induced stromal senescence surrounding tumors [23]. Oncogene-induced senescence (OIS) could stimulate senescence of CAAs by induction of SASP or gap junction-mediated cell-cell contact to enhance its own effects [24, 25]. For instance, numerous SASP factors such as TGF-β [25], IL-8, and CXCL1 [26], as well as some SASP pathways, including the NF-κB signaling pathway triggered by ROS [27] and cGAS–STING signaling [28], could intermediate the stimulation of paracrine senescence.
- CAAs are found to occur with brown/beige differentiation and have fibroblastic characteristics. Initially, a phenotypic switch from white adipose tissue to brown fat was discovered to exist in cachectic mice via overexpression of uncoupling protein-1 (UCP1) [29]. Then, UCP1 that is exclusively expressed in beige/brown adipocytes, PRDM16 that is a master regulator of brown adipocyte differentiation, gene expression for classical brown (MYF5, EVA1, and OPLAH) as well as beige (CD137/TNFRSF9 and TBX1) adipocyte markers is enriched in the samples of human and mouse breast cancer. Further, enrichment of beige/brown adipocytes in mouse models significantly promoted tumor development [13]. For the mechanisms, the exosomal contents and parathyroid Hormone-Related Protein (PTHrP) derived from tumor cells and IL-6 are found to promote WAT browning [12, 29–31]. Finally, the expressions of the fibroblastic biomarkers containing a-SMA and fibronectin are elevated in tumor-surrounding adipose tissues [13].
- CAAs prominently enhance multiple catabolic progresses and release multifarious high-energy metabolites including adenosine triphosphate (ATP), pyruvate, lactate, free fatty acids (FFAs) and glutamine [5, 31, 32]. An additional way for stromal adipocytes to interact with cancer cells is through the interchange of metabolites and amino acids between tumor cells and CAAs. Autophagy in stromal adipocytes could mediate lipolysis and release FFAs, a process that is subsequently utilized to fuel fatty acid oxidation (FAO) in breast cancer cells [12, 33]. In addition, the metabolic disorder of CAAs might be associated with changed immunoregulation, possibly by the generation of FFAs [34] or the consumption of immunomodulatory amino acids [35]. Moreover, ATP release increased in stromal cells that overexpressed UCP1 [36]; with respect to the mechanism, ATP release in adipocytes is dependent on Pannexin 1 and autophagy to induce lipolysis in adipocytes and promote macrophage migration [37, 38]. Finally, extracellular vesicles (EVs) are novel communicator between adipocytes and cancer cells [7, 12, 39, 40]. Adipocytes-derived EVs carry both proteins and substrates implicated in FAO to tumor cells, subsequently enhancing FAO and energy supply in melanoma cells to increase migration and invasion [7, 39]. Emphatically in obesity, EVs can incrementally transport fatty acids not fatty acid oxidation-related enzymes, subsequently store within lipid droplets in cancer cells.
- CAAs are also an important source of adipokines, chemokines, cytokines and exosomes, which could facilitate tumor growth and regulate treatment responses [5–7, 9]. CAAs secrete more chemokine (C–C motif) ligand 2 (CCL2), CCL5, interleukin-1β (IL-1β), IL-6, TNF-α, vascular endothelial growth factor (VEGF) and leptin, etc., which could facilitate the invasion of the tumor and immune escape [41]. For example, CCL2 and CCL5 can recruit macrophages and polarize them to M2-like subtype to form a specific structure called the crown-like structures (CLS). And the process is associated with malignant progression of breast cancer [42]. Additionally, IL-6 binds to its receptor IL-6R to regulating Janus kinase (JAK)/STAT3 signaling pathways, then stimulating the proliferation of tumor cells and the stemness of breast cancer stem cells (CSCs) [10]. Moreover, leptin is a multifunctional adipokine that has a regulatory effect in the immune system, as reviewed by Caitlin and et al. [43].
- PD-L1 expression is markedly elevated in CAAs to exert a tumor immunosuppression. First, there is a strong and specific expression of PD-L1 in the brown adipose tissue [44], suggesting that CAAs containing browning characteristics may overexpress PD-L1. Likewise, recent evidence indicates that the PD-L1 level is found to increase in mature adipocytes surrounding breast cancer. The activation of the antitumor function of CD8+ T cells by anti-PD-L1 antibody can be inhibited by adipocyte PD-L1 in vitro. In homologous breast tumor models, pharmacologic inhibition of adipogenesis selectively decreased the generation of PD-L1 in mouse adipose tissue and improved the anti-tumor efficacy of anti-PD-L1 or anti-PD-1 antibodies [45].

### The modulation of CAAs on innate immunity

CAAs have a profound impact on innate immunity. CAAs have various functions in the homeostasis and differentiation of innate immune cells, including supplying FFAs as energy and promoting the transformation among diverse metabolic pathways needed for differentiation. In addition, CAAs have a strong effect on the inflammatory mechanisms that constitute innate immunity. Once this interaction is disturbed, it will profoundly influence the development of cancer.

#### Neutrophils

Neutrophils have the shortest lifetime and are the most plentiful end-differentiated cells in the innate immune system. In the past decade, the effect and importance of neutrophils in obesity and cancer has become increasingly obvious [46, 47]. Importantly, obesity increases the infiltration of neutrophils in the adipose tissue, though the infiltrated neutrophils are far fewer than the macrophages. Two studies also showed that during the first week of a high-fat diet (HFD) treatment, the number of neutrophils increased, indicating that, as in the traditional immune responses, neutrophils may be one of the immune cells that migrate to the adipose tissues first in obesity [48, 49]. Likewise, gene knockout or pharmacological inhibition of neutrophils modifies insulin resistance induced by obesity and suppresses inflammation in adipose tissues [48]. Moreover, neutrophils accumulate in the peripheral blood of tumor patients, particularly those with advanced diseases, and the high ratio of circulating neutrophil-to-lymphocyte is a powerful predictive factor for poor prognosis in diverse tumors [46]. By contrast, the presence of intratumoral neutrophils, which are considered tumor-associated neutrophils (TANs), is an independent risk factor for short recurrence-free, cancer-specific, and overall survival in localized clear cell renal cell carcinomas (RCCs) [50]. Interestingly, obesity induces lung neutrophils to increase in normal mice, and the presence of primary tumors will further aggravate this disease. The increase in lung neutrophils transformed into elevated metastasis of breast cancer to this position, in a GM-CSF- and IL5-dependent manner [51]. Considerable evidence shows that CAAs in pancreatic ductal adenocarcinoma (PDAC) can secrete IL-1β to recruit TANs, further activating pancreatic stellate cells (PSCs). The interaction among TANs, adipocytes and PSCs facilitates tumor progression under conditions of obesity [52]. Meanwhile, CAAs release IL-8 to overexpress cell-adhesion molecules and induce neutrophil accumulation in breast cancer. High expression of IL-8 can increase the dissemination of cancer cells, while anti-IL-8 treatment exhibits suppression of angiogenesis at the primary tumor site and decreased dissemination of breast cancer cells [53]. Additionally, exocytosis of neutrophil-released ARG1 mediated by IL-8 leads to the depletion of arginine in the tumor microenvironment [54], thus hampering proliferation of T cells, and NO generated by NOS2 induces the suppression of CD8+ T cells [55].

Considering the metabolic factors, there are comparatively few mitochondria in neutrophils, and the energy needed for chemotaxis and function is provided mainly by glycolysis [56]. The inactivation of Prolyl hydroxylase 2 (PHD2) has been observed to result in the stabilization of HIF-1α, thereby elevating glycolytic flux and glycogen stores and facilitating abnormal responses in neutrophils [57]. Recently, a research study has demonstrated that even with the deprivation of glucose, glutamate and proline could retain the potential of immature low-density neutrophils (rather than high-density neutrophils) in tumors to form neutrophil extracellular traps (NETs), thus allowing their function to promote metastasis [58]. Neutrophil maturation is also controlled by metabolic state. Lipid droplets were accumulated in autophagy-deficient neutrophil precursors in which an energy crisis occurs, thereby preventing the transformation from glycolysis to oxidative phosphorylation (OXPHOS) which is needed for accurate differentiation. We speculate that the autophagy-deficient neutrophils fail to release FFAs through lipophagy (catabolizing lipid droplets to FFAs), thereby aggravating energy expenditure and resulting in differentiation defect, since supplying FFAs for autophagy-deficient precursor neutrophils completely restores differentiation. This research raises essential issues about the activation of lipophagy signaling pathways during differentiation of neutrophils and the degree of selective lipophagy [59]. Prominently, CAAs could release FFAs to promote neutrophil differentiation and export lactate and pyruvate to increase glycolytic flux in neutrophils, potentially maintaining the differentiation and function of TANs. Finally, ATP, a molecular currency for energy transfer, and its derivatives serve as the major biochemical constituents of the tumor microenvironment including heart function and immunomodulation [60, 61]. In answer to cytotoxic stimuli such as chemotherapeutic agents, cells undergo apoptosis, and then nucleotides derived from the cells are released into the extracellular space to facilitate the chemotaxis of phagocytes, phagocytosis and degradation of dead cells [62]. Various mechanisms, such as the exocytosis of vesicular ATP [63], the connexin-mediated secretion of cytoplasmic ATP via gap junction hemi-channels [64], the secretion via pannexin channels [65] or transmembrane transport via ATP transporters specific for the ATP-binding cassette family such as the cystic fibrosis transmembrane conductance regulator [66], contribute to the release of ATP induced by cell stress and cell death. ATP ligates a large proportion of subsets of the metabotropic P2YR and ionotropic P2XR purinergic receptor family [67]. ATP acts as a “find me” signal that facilitates the recruitment of neutrophils [68]. In the extracellular space, ATP and ADP are catabolized into AMP and adenosine by ectonucleotidases CD39 (also named NTPDase 1) and CD73 (5ʹ-NT), respectively, which are molecules located on the cell surface that are involved in the generation of adenosine [38]. Subsequently, extracellular adenosine could bind to any of four G protein-coupled purinergic type 1 receptors (adenosine receptor A1 (A1R), A2AR, A2BR or A3R), thereby stimulating the PKA signaling pathway through promoting the generation of cAMP mediated by adenylyl cyclase. Hypoxia is one of the most essential causes of the expression of CD39, CD73, A2AR and A2BR, and therefore has a determining effect on the outcome of purinergic signaling. Meanwhile, HIF-1α is upregulated in CAAs [12], suggesting that CAAs may overexpress CD39 and CD73 to produce adenosine by degrading ATP. Adenosine binding to A2ARs facilitates the production of anti-inflammatory IL-10 in neutrophils, thereby suppressing the responses of T and NK cells in the TME [69]. Likewise, adenosine inhibits the chemotaxis of neutrophils directly by binding to A3Rs or indirectly by weakening the release of chemoattractants from neutrophils [62]. In addition, adenosine also impairs their oxidative ability [70] and transendothelial migration [71]. In view of the duple effects of neutrophils on tumor development, the influence of neutrophil infiltration induced by CAAs and effect on tumor growth and metastasis is still unknown and needs more investigation.

#### Natural killer cells

Natural killer cells are innate immune cells derived from lymphoid. In response to infection, transformation or the presence of stressed cells, these cells generate IFN-γ, which triggers macrophages to polarize to a proinflammatory phenotype in turn. The basal metabolic rate of NK cells is low, with low levels of glycolysis and OXPHOS [72–74]. Such a low metabolic rate is enough to maintain acute NK cell responses, while glycolysis or OXPHOS cannot be stimulated by the generation of IFN-γ, cytokine stimulation or receptor ligation [72]. Nevertheless, this low metabolic rate is essential for acute NK cell responses. Inhibition of OXPHOS or glycolysis decreased the generation of IFN-γ significantly at these short time points, although degrees vary from the activation stimuli; receptor stimulation is much more sensitive to metabolic inhibition than cytokine stimulation is [72]. Intriguingly, continuous stimulation of NK cells leads to strong metabolic alterations that are needed for NK cell effector responses. Stimulating NK cells derived from mice or humans overnight with a combination of various cytokines leads to a greatly increased rate both of glycolysis and OXPHOS [73, 75]. Changes in the metabolic mechanism and structures of activated NK cells stimulated the upregulation in metabolic rates. The glucose absorption and flux elevate in activated NK cells via glycolysis, evidenced by elevated generation of glycolytic enzymes and related nutrient transporters [75]. In addition to the increases in OXPHOS rates and maximal respiratory capacity, mitochondrial mass also increases [76]. Whether fatty acids are utilized as a fuel source in NK cells has not been extensively studied, partially due to the lack of tools to detect fatty acid oxidation. Intriguingly, the accumulation of excessive fatty acids has been found to be harmful for metabolism and function of NK cells [77]. Therefore, glucose is the main energy source for NK cells, and glucose is utilized in the mitochondria and cytoplasm via aerobic glycolysis, thus driving the generation of OXPHOS and ATP through the citrate-malate shuttle (CMS) [76]. NK cell education (also called NK cell licensing) is one procedure to gain maturation in functions and self-tolerance. Cellular metabolic alterations have been proven to be related to the NK cell education process. Compared with uneducated NK cells, licensed NK cells derived from humans display elevated glucose transporter expression and higher glycolysis rates, instead of OXPHOS [78].

In the visceral adipose tissue of obese individuals, the number of NK cells increases, and the concentration of IFN-γ also increases [79]. The lack of NK cells reduces the accumulation of macrophages in white adipose tissue in the abdominal cavity and improves glucose tolerance in obese mice [80]. Providing mice with a high-fat diet upregulates the number of activated NK cells as well as the number of these cells that generate IFN-γ. The consumption of NK cells or weakened activation in high-fat diet mice reduces macrophages with proinflammatory phenotypes to accumulate in the white adipose tissue of the epididymis, along with the normalized glucose tolerance and insulin tolerance [81]. These results indicate that IFN-γ generated by NK cells might have an impact on the polarization of proinflammatory macrophages in epididymal white adipose tissue in obesity. Likewise, IL-6 and leptin derived from adipocytes could increase the PD-L1/NKG2D ligand level in cancer cells by activating the JAK/STAT3 signaling pathway, thereby decreasing the cytotoxicity of NK cells to tumor cells [82]. In obese mice, the antitumor responses of NK cells are impaired, and NK cells cannot control tumor growth [77]. Obese people, whether children or adults, have a decreased number of circulating NK cells compared to thin people, and these NK cells function abnormally, evidenced by less generation of IFN-γ, lower levels of granzyme B and perforin, and decreased cytotoxicity to tumor target cells [77, 83]. The functional abnormalities of NK cells have been connected to aberrant cellular metabolism in recent research studies [77, 83]. Compared with NK cells from lean mice or humans, NK cells derived from obese mice or humans cannot undergo metabolic reactions when stimulated by cytokines, and their metabolic rate is greatly reduced [77]. This metabolic dysfunction is related to lipid accumulation in NK cells driven by peroxisome proliferator-activated receptor (PPAR), resulting in changes in gene expression, decrease in MYC and mTORC1 signals, and reduced glycolysis and OXPHOS rates [77]. NK cells derived from obese mice and human lose the ability to kill tumor cells, partially due to the failure to form a synapse with target cells; the cytotoxic mechanism is not transmitted to the NK cell-tumor cell synapse [77]. The formation and maintenance of this NK cell–tumor cell synapse have been proven to be processes with energy expenditure [84]. NK cells derived from humans promote mitochondrial polarization to NK cell-tumor cell synapses, and after target cells participate, the mitochondrial membrane potential of the NK cells drops rapidly, in keeping with the rapid energy consumption [84]. However, CAAs release abundant FFAs and glycerine that may result in the accumulation of lipid in NK cells to impair functions of NK cells. In addition, CAAs can export high levels of lactate with a low pH, both of which could weaken the antitumor functions of NK cells [85]. Recently, a human research study demonstrated that a reduction of the intracellular pH of NK cells residing in the tumor-infiltrating liver was induced by the lactate generated by colorectal liver metastasis, resulting in mitochondrial dysfunction and apoptosis [86]. Therefore, signal changes and metabolic defects are major aspects of CAAs-induced NK cell dysfunction in the tumor microenvironment.

#### Natural killer T (NKT) cells

As a specialized subtype of T cells, NKT cells express both CD3 and NK1.1, which are cell markers of NK and NKT cells [87]. Therefore, NKT cells (CD3^+^ NK1.1^+^) can be distinguished from CD4 and CD8 T cells (NK1.1^−^ CD3^+^). In addition, NKT cells (CD3^+^ NK1.1^+^) could be distinguished from NK cells (CD3^−^ NK1.1^+^). These cells connect functions of innate and adaptive immune cells and could generate both type 1 (IFN-γ) and type 2 (IL-4 and IL-10) cytokines. In most situations, NKT cells cannot recognize peptide antigens presented by MHC I or MHC II molecules; however, the NKT cells recognize mainly glycolipid antigens presented by CD1d, a specialized antigen-presenting molecule many cells such as DCs, macrophages, adipocytes and hepatocytes express [88]. Nevertheless, a few subtypes of NKT cells also use MHC molecules instead of relying on CD1d. NKT cells are identified into two main subtypes according to their TCR sequences. The type I NKT cells (also called invariant NKT cells, classical NKT cells, or iNKT) have TCRs with an invariant α-chain (Va14-Ja18 in mice and Va24-Ja18 in humans), while the type II NKT cells (also called variant NKT cells, nonclassical NKT cells, or vNKT cells) have more diverse TCR sequences. Therefore, both iNKT and vNKT cells are absent in CD1d^−/−^ knockout mice, while only iNKT cells are absent in Jα18^−/−^ knockout mice. Various cytokines, including IL-4, IL-13, IFN-γ and TNFα, are generated by NKT cells, thereby mediating either T helper 1 (Th1) or Th2 responses [89]. In colorectal cancer (CRC), the increase in tumor metastasis to the liver was lacking natural killer T (NKT) cells [90]. Moreover, probiotics can mediate the conversion of primary to secondary bile acids, thereby increasing the expression of CXCL16 in liver sinusoidal endothelial cells. Probiotics can also further exert antitumor effects in a liver-selective manner, upregulating hepatic CXCR6+ NKT cells and increasing the generation of IFN-γ under antigen stimulation [91]. The number of iNKT cells in the adipose tissue is inversely proportional to the degree of obesity [92]. The number of these cells decreased when a high-fat diet was used, which was reversed by the removal of high-fat feeding in mice [93]. Recent research has demonstrated that to explore the roles of NKT cells in obesity, mice with CD1d^−/−^ and Jα18^−/−^ knockout exhibited impaired glucose and insulin tolerance, as well as elevated infiltration of proinflammatory macrophages in adipose tissue [92, 93]. Another research study demonstrated that β2-microglobulin (B2M) knockout mice showed a deficiency of NKT cells and were insensitive to insulin resistance induced by obesity, indicating that NKT cells might participate in the progression of insulin resistance induced by obesity [94]. Hence, CAA may secrete several regulators or decrease CD1d to impair NKT cell functions. The effect of NKT cells on the CAAs-induced tumor progression is not yet completely understood and needs further investigation.

#### Macrophages

Macrophages function as scavengers to regulate the immune response to pathogens and maintain tissue homeostasis. As one complex subtype of macrophages, adipose tissue macrophages (ATM) express various surface markers and have distinctive anatomical positions [95]. Moreover, two major phenotypes of ATMs are the classical polarized M1 macrophages and alternatively polarized M2 macrophages [96]. For inflammation, M1 macrophages generally secrete proinflammatory cytokines (such as TNF-α, IL-6), whereas M2 macrophages have an anti-inflammatory effect through excreting IL-10 [97]. Although tumor-associated macrophages (TAMs) cannot be divided into M1 and M2 subpopulations simply, they often display an M2-like phenotype and promote tumor growth through stimulating immune-suppression [97]. Importantly, reprogramming of macrophages from one phenotype into another partially explains the diversity of macrophages [98]. Therefore, it is crucial for obesity-associated tumors to understand the heterogeneity and plasticity of macrophages. Crown-like structures (CLSs) are termed a configuration where macrophages surround a dead or dying adipocyte [99]. The number and density of CLSs are positively associated with high BMI, large adipocyte size, postmenopausal status and insulin resistance in obese patients, indicating that the CLS has a pathophysiologic effect on adipose tissue inflammation [100, 101]. The pathological upregulation of CLS in mammary adipose tissue is associated with an inferior prognosis in breast cancer patients [100]. Considerable evidence indicates that, according to the different environmental cues, ATMs switch their transcription process from “steady state” to “polarization”, into an inflammatory state, or vice versa [102].

The differentiation, mobilization, polarization, and antitumor response of macrophages can be modulated by metabolism. Macrophage metabolism is now recognized as a complicated accurately controlled program that affects and/or is affected by diverse characteristics of tumor cells and the tumor microenvironment. First, the activity of macrophages against pathogens as well as tumor cells needs aerobic glycolysis to supply energy. The stimulation of lipopolysaccharides (LPS) induces M1 polarization of macrophages, leading to elevated aerobic glycolysis [103]. In addition, glycolysis promotes the carbon influx into the oxidative pentose phosphate pathway (PPP), which generates NADPH to produce reactive oxygen species (ROS) by NADPH oxidases. The generation of ROS is crucial for the phagocytic activity of M1 macrophages. Inhibiting aerobic glycolysis through activation of pyruvate kinase M2 (PKM2) or inhibiting pyruvate dehydrogenase kinase 1 (PDK1) reduces the M1 polarization of macrophages induced by LPS [104]. In addition to M1 polarization, glycolysis is also essential for M2 polarization of macrophages [105], as glycolysis has a vital effect on the generation of cytokines by M2 macrophages stimulated by LPSs [106]. In addition, through stimulating the expression of the major glycolytic enzyme PFKFB3, soluble factors such as hyaluronan fragments derived from a tumor could promote glycolysis in TAMs [107]. Second, fatty acid oxidation (FAO) acts as the essential energy source to promote the M2 polarization of macrophages. For FAO, triacylglycerol substrates are internalized by M2-polarized macrophages through the scavenger receptor cluster of differentiation 36 (CD36), which later undergoes lipolysis via lysosomal acid lipases [108]. The IL-4 stimulation induces M2 polarization of macrophages, thereby promoting fatty acid uptake and oxidation and enhancing mitochondrial biogenesis [109]. Inhibition of FAO in TAMs facilitates the antitumorigenic differentiation of TAMs and suppresses tumor growth [110]. The mechanism through which FAO is promoted in macrophages has been well established. Activation of peroxisome proliferator-activated receptor-gamma (PPAR-γ) and downstream of the PPARg-coactivator-1β (PGC-1β) induces FAO and mitochondrial biogenesis transcriptionally [109, 111]. In vitro and in vivo, tumorigenic polarization or M2 polarization of macrophages requires PPAR-γ [109, 110]. In addition, PGC-1β is necessary and sufficient to induce M2 polarization of macrophages [111]. Therefore, FAO supports protumorigenic polarization of macrophages.

Given that macrophages are crucial factors in most chronic inflammation cases, we speculate that inflammation induced by obesity is also stimulated by macrophages. In general, there is evidence showing that obesity elevates the number of ATMs in animals and humans [112]. This study also found that weight loss leads to a decrease in the number of ATMs, and this decrease is often accompanied by an improvement in insulin resistance (such as that induced by TZD treatment). The most important alternation induced by obesity in ATMs is the increase of the number of triple-positive (CD11b^+^ F4/80^+^ CD11c^+^) ATM populations [102]. Numerous research studies tried to classify ATMs based on the M1/M2 system. At present, the general consensus in this field is that obesity will induce complete polarization of ATMs from M2 phenotype to M1 phenotype. Nevertheless, flow cytometry analyses reveal that both CD11c (M1 marker) and CD301 (M2 marker) exist meanwhile in ATMs [102], indicating that a single ATM can simultaneously have both M1 and M2 features. Moreover, the M2 phenotype but not M1 phenotype is related to BMI in humans [113], and ATMs derived from obese humans displayed the genetic characteristics of TAMs [114]. These findings indicate that macrophage might mediate obesity-induced tumor metastasis and immune escape.

As described above, adenosine is prone to be accumulated in a tumor-associated adipose microenvironment (TAME), contributing mainly by the secretion of CAAs. Upon binding to A2A receptors, adenosine reduces the classical polarization of macrophages, whereas M2 polarization is induced when adenosine binds to the A2B receptor. Adenosine promotes the recruitment of cultured monocytes to tumors [115]. The ectonucleotidases CD39 and CD73 could also be produced in TAMs, which could further catalyze the generation of adenosine and transmit signals to downstream adenosine receptors. Therefore, because of the upregulated cytotoxicity of NK and T cells, A2A receptor deficiency in myeloid cells suppresses the growth and metastasis of melanoma [69]. In addition, the knockout of A2A receptors in myeloid cells reduces M2 polarization, while enhancing M1 polarization in TAMs [69]. Specifically, the suppression of T cell proliferation mediated by TAMs could be reversed by inhibiting CD39 or CD73 [115]. Thus, metabolic changes that promote the accumulation of adenosine in the TAME cause tumor immunosuppression by promoting macrophage polarization to the M2 phenotype.

In the TAME, lactate could modulate the signaling functions and polarization of M2 macrophages. Lactate is the final product of aerobic glycolysis in some types of cells such as CAAs [12, 31, 32]. Lactate can also regulate the polarization of macrophages directly [116]. Lactate is competent to stimulate expression of VEGF and markers of M2 polarization such as Arg1, Fizz1, Mgl1, and Mgl2. G-protein-coupled receptor 132 (Gpr132) on macrophages mediates the sensation of lactate by M2 polarized macrophages, and the metastasis of breast cancer is suppressed with the loss of Gpr132 in mice [117]. Furthermore, the decrease of Gpr132 expression is related to the improvement of metastasis-free survival in breast cancer patients. Therefore, the level of lactate in TAME modulates the signaling functions and M2 polarization of macrophages.

#### Myeloid-derived suppressor cells (MDSCs)

MDSCs are consisted of a heterogeneous subtype of immature myeloid cells that are derived from the common myeloid progenitor [118]. There are two subsets of MDSC, monocytic (M-MDSC) and polymorphonuclear (PMN-MDSC). MDSCs exert immune-suppressive effects to promote tumor growth and inhibit T cell activation through impairing immunity by elevating arginase-1, nitric oxide (NO), and ROS [118]. In obese individuals, the infiltration of immune suppressive MDSCs was upregulated [119]. Leptin, an over-expressed adipokine in obese adipose tissue, induces the accumulation of MDSCs in peripheral serum and in tumors [120]. Polyunsaturated fatty acids (PUFAs) promoted the differentiation of MDSCs and elevated the immune suppressive ability through JAK-STAT3 signaling pathway. This pathway further upregulated NADPH oxidase to facilitate the generation of ROS [121]. Instead of glycolysis, MDSCs rely on fatty acid oxidation to supply energy. Tumor-derived colony-stimulating factor (CSF) promoted the lipid metabolism and fatty acid oxidation in MDSCs through STAT3 and STAT5. STAT3 and STAT5 signaling pathway increased the synthesis of lipid transport receptors resulting in an elevated lipids uptake. The enhanced lipid metabolism upregulated the ability of MDSC to suppress T cell activation [122]. Taken together, adipokines and lipids which were derived from adipocytes in the TME facilitated the infiltration and T cell suppression of MDSCs.

### The modulation of CAAs in adaptive immunity

The main adaptive immune response to kill tumor cells begins with recognizing tumor antigen via antigen-presenting cells (APCs), which further present tumor-associated antigens by MHC II molecules to the T cell receptors (TCRs) of CD4 helper T (Th) cells. Ultimately, these processes result in activation of T effector cells (Teffs), which could finally result in the eradication of cancer cells. CAAs in TAME exert direct or indirect effects on the process of adaptive anti-tumor immunity to promote tumor immune evasion. Most directly, CAA upregulated PD-L1 binds to PD1 on the surface of CD8+ Teff, subsequently suppressing antitumor functions of CD8+ T cells [45]. In addition, CAAs could release immunomodulatory metabolites and secrete adipokines such as leptin to mediate the differentiation of adaptive immune cells. Hence, the CAAs-mediated immune tumor microenvironment strongly influences tumor progression.

#### Dendritic cells (DCs)

DCs have a crucial effect on the switch from innate immunity to adaptive immunity through presenting antigens by MHC II molecules to the TCRs of Th cells [123]. Likewise, various cytokines are generated by DCs, which function to promote the maturation and/or activation of other immune cells. For instance, DC-derived IL-12 stimulates the differentiation of naïve T cells into Th1 T cells, while IL-15 released from DCs has a vital impact on the proliferation and activation of CD8 T cells and NK cells. CD11c is the main surface marker of DC cells analyzed by flow cytometry, while CD80, CD83 and CD86 are often utilized together with other markers [124]. Two key subtypes of DCs in the TME, plasmacytoid DCs (pDCs) and conventional DCs (cDCs) are derived from the common DC progenitor (CDP) but have different morphologies and functions [125]. The pDC was originally reported to generate type I interferons in answer to viral infections. In the TME, pDCs promote the generation of Sema4A and IDO to support Tregs functions, thereby playing a role in immunosuppression and facilitating the progression and metastasis of tumors [126]. According to the different functions and surface markers, cDCs could be further classified into two subtypes, cDC1 and cDC2 [125]. cDC1 expresses XCR1 and CD8α in the lymphoid organs or CD103 within peripheral tissues and needs the transcription factors IRF8, BATF3, and ID2 for development. In the meantime, cDC2 expresses CD11b and SIRPα and needs the transcription factors IRF4, ZEB2 and Notch2 for the development. cDC1 plays an important role in eliciting an anti-tumor CD8+ T cell response mediated by MHC-I and supports T cell effector functions by releasing IL-12, while cDC2 appears to be involved in the activation of CD4+ T cells mediated by MHC-II [127]. Moreover, cDC1 recruits CD8+ T cells via the generation of CXCL9 and CXCL10 [128]. These observations suggest the important effects that cDC1s have on the control of tumor growth. Regarding immunotherapy, cDC1s play an important role in facilitating the anti-tumor effects of the PD-1 blockade and efficient adoptive T cell transfer therapy [128]. Furthermore, in mouse tumor models, the immune status determines the vaccination efficiency of cDC1 or cDC2 vaccine. Inoculation of cDC1 can enhance the anti-tumor performance of CD8+ T cells, while vaccination of cDC2 can enhance the differentiation of TH17 cells (IL-17-expressing T cells) and promote the polarization of TAMs to an M1-like phenotype with anti-tumor activity [129]. The genetic markers of cDC1 are positively associated with the survival of human cancer patients with different tumor types, including breast cancer, head and neck squamous cell carcinoma, and lung adenocarcinoma [130].

Because the main function of DCs is to present antigens in adaptive immunity, and inflammation induced by obesity is generally thought to be caused by innate immunity, the role of DCs in inflammation induced by obesity has not yet been comprehensively demonstrated. Recently, two research studies, both of which revealed that obesity induced the increase of the infiltrated DCs in adipose tissue [131, 132], concentrated on the effects of DCs on the progression of insulin resistance induced by obesity. One study demonstrated that there were three DC subtypes, classified as myeloid, CD4+, and CD8+ DCs, in adipose tissue, and the number of those CD103 DCs decreased under conditions of obesity [131], which played a vital role in the differentiation of regulator T (Treg) cells in small intestinal lamina propria [133]. Intriguingly, the DCs derived from adipose tissue of obese animals and humans could stimulate the differentiation of Th17 cells in vitro [131]. The differentiation of Tregs and Th17 cells usually counterbalance each other, and obesity induced the decrease of Treg numbers. Th17 cells have not yet been identified in adipose tissues (ATs) from lean or obese animals. Therefore, it would be interesting to explore whether CD103 DCs could modulate the inflammation induced by obesity in AT through modulating the balance of Treg and Th17 cell differentiation in vivo. In addition, the other study demonstrated that the loss of DCs in Flt3–1−/− knockout mice improved insulin resistance induced by obesity, as well as reduced the numbers of ATMs and liver macrophages, which were reversed by the reconstitution of DCs [132]. These results indicate that adaptive immunity might also be involved in promoting inflammation induced by obesity. Hence, it will be a promising direction to investigate whether DCs have an impact on the progression of obesity-induced inflammation by facilitating adaptive immunity.

Activated DCs meet their energy requirements via glycolysis and the pentose phosphate pathway (PPP). Once ligated to TLRs, the uptake of glucose and generation of lactate are immediately elevated in DCs [134]. Glycolysis provides substrates for the PPP and TCA cycle to generate NADPH and mitochondrial citrate, respectively, which are then transported to the cytoplasm to fuel FAS [134]. The citrate flux into FAS is necessary for the extension of the endoplasmic reticulum (ER) and the Golgi apparatus in DCs [134]. The unique utilization of citrate in DCs is considered to be a key event that supports the activation of maturation and special biological activities in DCs [134]. The proinflammatory signatures of DCs are generally enhanced by glucose metabolism and FAS, but actually, some research studies have shown that the activation of T cells induced by DCs are upregulated through the inhibition of these pathways [135]. Mechanistically, glucose restriction in DC-T cell synapses will inhibit the mTORC1/HIF-1α/NOS2 signaling pathway in DCs, thereby reducing the speed of glycolysis [135]. Moreover, interferon-I could upregulate the mitochondrial activity and FAO that promote pDC maturation [136]. In contrast, in mouse tumor models and patients with cancer, the capability of cDCs to process tumor antigens and activate T cells effectively will be weakened by the accumulation of lipids, which is caused by FAS activation and lipid uptake through the increased expression of Msr1 [137]. Potentially, pDCs in TAME may increase uptake of FFAs and accumulate lipids, thereby impairing their antigen-presentation function and inactivating Teff cells. In addition, DCs could adjust their activity and function through sensing extracellular metabolites such adenosine/ATP [138] and lactate [139]. Therefore, adenosine derived from CAAs binds to adenosine receptor on surface of DCs, then suppresses proinflammatory IL-12, increasing anti-inflammatory IL-10, activating NF-κB and PKA-Epac pathways, and consequently inhibiting activation of T cells [140]. Likewise, lactate in TAME is sufficient to suppress IFN type-I and has an effect on adaptive function, enhancing antigen degradation and decreasing cross-presentation. Finally, lactate-induced DCs failed to trigger antitumor responses [141]. The lactate receptor GPR81 in colonic dendritic cells has a crucial effect on inhibition of colonic inflammation and recovery of colonic homeostasis [142].

#### Regulatory T cells (Tregs)

Regulatory T cells (Tregs) prevent obvious immune responses and autoimmunity and abnormally accumulate in certain tumors to suppress antitumor immunity and participate in the formation of an immunosuppressive microenvironment. Tregs are a subpopulation of the CD4+ T cells. Characterized by highly expressed CD25 (IL-2 receptor α-chain) and the transcription factor fork head box P3 (FoxP3), Tregs are involved in suppressing immune responses [143], and there are two different subtypes of Tregs [144]. One subset forms along thymopoiesis that originates from the differentiation of naïve T cells upon TCR stimulation or from functionally mature precursors that express CD25, thus forming a natural subset named thymus-derived Tregs (tTregs). tTregs can be recruited to the peripheral locations to play a role in immunosuppression. The other subset, named peripheral-induced Tregs (pTregs), is produced from mature CD4+ T cells in the peripheral lymphoid organs upon the stimulation of certain antigens or suppressive cytokines, including TGF-β. The suppressive ability of pTregs varies according to the local microenvironment and usually depends directly on the generation of cytokines in different disease situations [145]. Upon activation, Tregs can further differentiate into different effector subtypes, including memory-like and tissue-resident Tregs that exert crucial functions in nonlymphoid organs [146].

Increasing evidence has shown that there are highly infiltrated Tregs in a variety of tumor types in humans and mice, including skin [147], pancreas, breast [148], and ovarian [149] tumors. The infiltrated level of Tregs is usually higher in advanced cancers (stage III or IV). The infiltration of Tregs into the tumor was negatively associated with survival [150], while a decreased infiltrating CD8+ T cell:Treg ratio in tumors was related to poor prognosis [151]. Therefore, it is currently described that Tregs in tumors promote the progression, invasion and metastasis of the tumor [152]. Tregs inhibit the immune responses of T cells and activities of antigen-presenting cells such as DCs and macrophages. For instance, effector T cells could be killed by Tregs in tumors via the FasL-Fas signaling pathway and cytotoxicity mediated by granzyme B and perforin through direct cell–cell contact [153]. Acting as a sink for IL-2, Tregs could neutralize IL-2 in the microenvironment, as directly highly expresses CD25, the alpha chain of the IL-2 receptor. The depletion of IL-2 leads to the survival and metabolic destruction in target cells [154]. In addition, anti-inflammatory cytokines such as IL-10, IL-35, and TGF-β could be released from Tregs to avoid the activation of innate and adaptive immune cells and to facilitate tumor growth [155]. Furthermore, TGF-β could induce Tregs to accumulate in tumors by facilitating the differentiation of naïve CD4+ T cells to Tregs [156]. Tregs could also release vascular endothelial growth factor (VEGF) to facilitate neovascularization via cooperation with TGF-β [157]. The constitutive expression of CTLA-4, a coinhibitory molecule, is a major characteristic for Tregs [158]. The combination of CTLA-4 with CD80/CD86 on APCs induces indoleamine-2,3-dioxygenase (IDO) to consume essential amino acids, thereby suppressing the proliferation of target cells [159]. Except for TGF-β, other mechanisms have also been involved in promoting Treg accumulation in TME. For instance, the CC chemokine ligand 22 (CCL22), which is secreted by cancer cells, facilitates the recruitment and maintenance of CC-chemokine receptor 4 (CCR4+) Tregs in the TME [149]. Hypoxia promotes the expression of CCL28, resulting in abnormal accumulation of Tregs via the CCL28-CCR10 pathway in ovarian cancer cells [157]. Cancer cells can also generate immune-suppressive cytokines, including VEGF and IL-10, to facilitate the differentiation of pTregs and expansion of natural Tregs (nTregs) through stimulating the generation of abnormally functional antigen-presenting cells [160].

The association between obesity and its diverse complications involves chronic low-grade inflammation in adipose tissue (AT) [161], which is facilitated by various proinflammatory cytokines secreted by adipocytes and various immune cells such as macrophages [162], resulting in metabolic abnormalities and type 2 diabetes. In the lean conditions, considering Tregs as an example, fewer immune cells, most of which exhibit an anti-inflammatory phenotype, infiltrate into the AT. An AT-resident Treg population has been shown to account for a relatively large proportion of anti-inflammatory immune cells in AT [163]. Notably, Tregs were found to accumulate only in abdominal adipose tissue of lean mice rather than obese mice, which showed a particular phenotype with the ability to regulate the insulin sensitivity of adipocytes by restricting adipose tissue inflammation [163]. The comparative Treg number among CD4+ T cells was much higher than in other tissues of healthy individuals, and the number decreases in obesity [164]. PPAR-γ is identified as the major molecular mechanism of the formation of AT Tregs. PPAR-γ stimulates the differentiation of adipocytes and promotes the progression, infiltration, and phenotype of these cells [163]. The lack of PPAR-γ in Tregs reduced the number of AT Tregs and specifically altered the transcription characteristics of these cells in obese mice, which indicated that PPAR-γ is the main regulator of AT Treg phenotype, and obesity may affect AT Tregs by regulation of PPAR-γ [165]. However, the mRNA level of PPAR-γ in obese mice was comparable to the mRNA level of PPAR-γ in lean mice [164], indicating that the dysregulated genes in obesity have nothing to do with the downregulated PPAR-γ expression. We speculate that alterations of AT Tregs in obesity may be induced by the posttranslational modification of PPAR-γ as well as by the absorption and generation of PPAR-γ lipid ligands. Further research concentrated on AT Tregs has shown that their development proceeds in the following way: Tregs induced in secondary lymphoid organs express low levels of PPAR-γ, and then differentiate into Tregs with high PPAR-γ expression when migrating into adipose tissue, which depends largely on the unique TCR activation such as IRF and BATF95 and specific signals such as IL33-ST2 [166]. In general, although Tregs are involved mainly in certain transcription processes to maintain their major functions, in different tissue environments, to ensure their development and unique functional applications, a high degree of plasticity is still required. Some factors lead to the plasticity directly, including metabolic signals responding to the availability of nutrients and the systemic metabolic state. These observations also indicate that the regulation of lipid metabolism and Tregs in adipose tissue may selectively regulate the inflammatory state in AT by regulating the immune response of AT Tregs.

The abnormalities of energy metabolism, such as elevated aerobic glycolysis and lipolysis in TAME, help cancer cells to meet their energy demands for proliferation. However, we think that fatty acid oxidation (FAO) and oxidative phosphorylation (OXPHOS) provide most of the energy for the differentiation and function of Tregs [167, 168]. First, the increased generation of fatty acids in CAAs might greatly elevate lipid availability in the TAME. Because the differentiation and survival of Tregs rely mainly on FA uptake and catabolism [169], intratumoral Tregs may selectively participate in lipid metabolism pathways to ensure their accumulation in tumors. A recent research study showed that the expression of some FA binding proteins in intratumoral Tregs is higher than that in Tregs in peripheral blood and normal tissues of breast cancer patients [170]. In addition to FAs, the augmented lactate levels in the TAME might potentially supply metabolites for Tregs in tumors, since lactate dehydrogenase (LDH) catalyzes the reversible transition of lactate to generate pyruvate and NADH. Notably, monocarboxylate transporters (MCTs) have been demonstrated to mediate the import of lactate from an extracellular matrix into Tregs to maintain their differentiation and survival in vitro [168]. Importantly, increased uptake of lactate could weaken aerobic glycolysis and enhance engagement of OXPHOS, which is a metabolic preference of Tregs. Hence, the elevated activity of aerobic glycolysis in CAAs forms a lactate-enriched microenvironment which provides enough lactate for the survival of Tregs and facilitates the immunosuppressive response of Tregs in tumors. Actually, the survival of Tregs in a lactate-enriched environment has recently been proved to be ensured by their specialized metabolic preferences [171]. Nevertheless, whether lactate and FA metabolism play a role in the accumulation of Tregs in tumors, as well as the underlying mechanisms, needs further investigation. Ultimately, an elevated level of CD39 in Treg cells facilitates degradation of the ATP secreted by CAAs into adenosine. Furthermore, adenosine facilitates the differentiation of immunosuppressive Treg cells. Upon A2AR activation, naïve CD4+ T cells were differentiated into CD4+FOXP3+ T cells, and expression of A2AR on Tregs elevates their immunosuppressive functions [172].

### Effector T cell and memory T cells

Effector T (Teff) cells act as an important component in the adaptive immune system, serving as both coordinators and effectors of immunity. Glycolysis is utilized in activated Teff cells to support proliferation and maintain the function as effectors. Recently, a research study demonstrated that 10% of the cellular carbon in activated Teff cells comes from glucose, while another 10% comes from glutamine [173], indicating that Teff cells cultured in vitro mainly utilize aerobic glycolysis and glutaminolysis to produce ATP and maintain redox balance to facilitate rapid proliferation instead of biomass accumulation. This distribution of metabolites can redirect lipids and other amino acids such as DNA nucleotides and membrane lipids to generate biomass to facilitate cell division and Teff cell function [174]. As T cells cultured in vitro are supplied with excessive glucose, glutamine, and other amino acids, further investigation is needed to determine whether T cells mainly utilize glucose and glutamine for biosynthesis in vivo and distribute those metabolic substrates for the production of biomass similarly. In addition, substrate utilization in Teff cells is affected by growth factors, strength of TCR activation, stimulatory signals, and other immune cells. For instance, the costimulation of CD28 with TCR promotes glycolytic metabolism and prevents anergy [175]. Likewise, various signals could affect the utilization of substrates and Teff cell functions [176]. Importantly, metabolism could directly influence the epigenetic characteristics of cells by changing histone activities and DNA modification enzymes and supplying the metabolites necessary for epigenetic modification [177].

Memory T cells are a subpopulation of T cells that can “remember” previously encountered homologous antigens. CD8+ central memory T cells (Tcms) show high expression of the L-selectin (CD62L) homing receptor on the protein level, as well as a large amount of FAO and spare respiratory capacity (SRC) in mitochondria, accompanied by elevated expression of carnitine palmitoyltransferase 1α (CPT1α), which is one of the rate-limiting enzymes for FAO [178]. SRC was downregulated in vitro by the pharmacological inhibitory effect of etomoxir on CPT1a, indicating that the enhancement of SRC in Tcm cells is mostly induced by FAO mitochondria [178]. The particular knockout of von Hippel-Lindau (VHL), the negative regulator for HIF-1α, induces the constitutive activation of HIF-1α and increases the generation of granzyme B and TNF-α [179]. Compared with lymphoid-resident CD8+ Tcm cells, CD8+ T cells with VHL deficiency that have undergone constitutive glycolysis were capable of producing effector memory T cells (Tems), featuring low expression of CD62L [180]. Furthermore, CD8+ Tem cells lacking VHL showed considerably increased basal and maximal ECAR, as well as mediated secondary expansion depending on glycolysis [180]. In general, these observations suggest that in addition to effector cells, glycolysis could support long-lived CD8+ Tem cell functions, while mitochondrial SRC seems to be able to characterize lymphoid-resident CD8+ Tcm cells rather than Tem cells. How the substrates of mitochondrial FAO are produced in different memory subsets is still largely unknown. Although highly dependent on FAO, CD8+ Tcm cells did not upregulate their FA uptake from the extracellular matrix, instead relying on cell intrinsic lipolysis of triacylglycerides (TAGs) mediated by lysosomal acid lipase (LAL) to provide energy for FAO [178]. It is still unclear why this ineffectual cycle of FA synthesis and oxidation happens in CD8+ Tcm cells. A reasonable hypothesis is that the cycle might be needed to maintain redox balance, supply metabolic substrates and keep normal mitochondria characterized by continuous glycolysis and lipogenesis activities, which remains to be explored further. Overall, these findings indicate that effector differentiation of CD8+ T cells is dependent on de novo synthesis of lipids, while the formation and function of CD8+ Tcm cells need both lipid synthesis and oxidation.

Obesity upregulates the number of CD8+ T cells in adipose tissue and induces the expression of IFN-γ and granzyme B [181–183]. A study further demonstrated that the CD8+ T cell expenditure could improve insulin resistance caused by obesity, which is related to the specific reduction of CD11c+ ATMs, while the number of CD11c- ATMs remains unchanged [181]. Moreover, CD8+ T cell expenditure suppressed the increase of proinflammatory cytokine expression induced by obesity, including IL-6 and TNFα. In addition, splenic CD8+ T cells were used to reconstitute CD8a−/− KO mice fed with a HFD, which aggravated insulin resistance induced by obesity. This process was correlated with the elevated number of CD11c+ ATMs, no alterations in CD11c− ATMs, as well as upregulated genetic expression of IL-6 and TNFα in AT. As these findings strongly indicate that CD8+ T cells stimulate the infiltrated number of ATMs and that the alterations in the number of AT CD8 T+ cells were anterior to the increase in the ATM numbers in obesity, we speculated that the recruitment of ATMs was induced by AT CD8+ T cells under conditions of obesity, thus inducing insulin resistance development. Intriguingly, they also demonstrated that compared with the lean controls, AT derived from mice fed on an HFD elevated the proliferation of cocultured splenic CD8+ T cells in vitro, indicating that AT CD8+ T cells might clonally expand in obesity, which suggests a crucial mechanism through which obesity induces the inflation of CD8+ T cells in adipose tissue.

CD8+ Teff cells have an important effect on cancers. All processes of CD8+ Teff cell functionality and longevity such as clonal expansion, contraction, memory formation, and ‘exhaustion’ are maintained by different metabolic situations, and the switch from one to another is strongly modulated in cancer immunotherapy. Infiltrated CD8+ Teff cells in tumors experience metabolic depletion in the acidic, FFA-enriched, oxygen-and nutrient-deficient TAME. First, the resting naïve CD8+ T cells maintain low metabolic requirements and are dependent on OXPHOS to produce ATP, in which nutrients are utilized mainly for its survival and homeostasis [184]. Once activated, both OXPHOS and glycolysis in CD8+ T cells are engaged to achieve the bioenergy and biosynthesis requirements related to the proliferation during the clonal expansion [185]. In this aspect, effector T cells and cancer cells have similar metabolic characteristics, including increased uptake of glucose and enhanced glycolysis processes [186]. Increased expression of glucose transporter 1 (Glut1) on cell surface is an early issue during T cell activation [187]. After TCR activation and costimulation, the PI3K-Akt pathway and the key metabolic regulators mTOR and c-myc upregulate the glucose uptake and glycolysis closely in mouse CD8+ T cells [184, 188]. Likewise, the activation of CD8+ Tm cells requires increased glucose metabolism to promote their proliferation and following Teff cell responses [189]. In addition, glycolysis and OXPHOS can be involved in supporting the important metabolic requirements of T cells after activation [190]. Although aerobic glycolysis plays an important role in gaining activity or function, but not so important for the proliferation and survival of T cells [190], mitochondria are necessary for the activation of T cells, partially by the production of reactive oxygen species (ROS), which results in the activation of the nuclear factor of activated T cells (NFATs) and the generation of IL-2, which has an impact on T cell function [191]. However, the massive production of lactate by CAAs is proven to suppress effects or functions as well as the cytotoxicity of CD8+ T cells [85]. Similarly, adding cultured supernatants of EL-4 T lymphoma cells to mouse CD8+ T cells to reduce the extracellular pH will significantly upregulate its cytotoxicity, which can then be reversed by neutralizing acidic media [192]. In addition, PD-L1 expressed in CAA suppresses the differentiation of human effector CD4+ T cells through suppressing glycolysis and facilitating FAO by elevating the expression of CPT-1α [193]. Therefore, a PD-1/PD-L1 blockade could downregulate aerobic glycolysis in some cancers, resulting in the restoration of Teff functions. Considering the large amount of ATP released by CAAs and the overexpression of CD39 and/or CD73 in other stromal and/or immune cells in TAME, we speculated that TAME may have the potential to enrich adenosine, the accumulation of which suppresses the cytotoxicity of CD8+ T cells [194]. Therefore, metabolic reprogramming of CD8+ T cells might supply a useful therapeutic strategy for cancer treatment.

### Targeting CAAs for clinical benefit of tumor immunotherapy

CAAs exert immunosuppressive functions during cancer development, and thus target CAAs would represent a tempting and promising therapeutic addition for anti-tumor immune intervention. However, the diversity of CAA functions and subsets brings multiple barriers and challenges. Especially, lacking specific cell surface markers of CAAs restrains the direct detection, thus targeting CAAs precisely is difficult without damaging normal tissues. Along with the further understanding about CAAs in the tumor immune microenvironment, attention to CAA-targeted therapy continues to increase. Patients may benefit from targeting CAA-associated effectors or reprogramming of CAAs into a normal phenotype.

Making CAAs more ‘normal’ has been an attractive approach. The reactivation of PPAR-γ in CAAs provides an example of this strategy. For example, propranolol, as a β-adrenoreceptor antagonist, could partially reverse tumor-induced activation of CAAs by elevating PPAR-γ expression [31, 32]. Furthermore, propranolol reduced intratumoral mesenchymal polarization and recruited neutrophils, natural killer cells, and dendritic cells at the tumor site in early-stage surgically resectable breast cancer [195]. Propranolol then strongly improved the efficacy of an antitumor STxBE7 vaccine by enhancing the frequency of CD8+ T cells infiltrating the tumor [196]. Moreover, the PPAR-γ agonist rosiglitazone combined with MEK inhibitors could impair the trans-differentiation and functional alteration of adipocytes forced by breast cancer cells [16]. Likewise, rosiglitazone facilitated the increase of infiltrated CD3+ T cells in the tumor while inhibiting the late accumulation of CD11b+ and Gr-1+ myeloid cells to significantly retard tumor growth [197]. Rosiglitazone limited the accumulation of early MDSCs and Tregs but increased circulating CD8+ T cells and intratumoral CD4+ and CD8+ T cells to change tumor-associated immunosuppressive mediators, thereby enhancing the effect of gemcitabine [198]. Hence, it will be a promising direction to normalize tumor-promoting CAAs or to change their phenotype for exploring anticancer targeted therapies.

In practice, normalization or reprogramming of CAAs is not necessary to obtain clinical benefit, as clinical benefit can be achieved by blocking signals derived from the CAAs. A better understanding of the metabolic pathways that are differentially utilized in cancer cells and stromal cells can provide a new perspective for the progression of therapies that could facilitate anti-tumor immunity. For instance, fatty acid metabolism is engaged in melanoma and ovarian tumors, so these tumors could benefit from the inhibition of the fatty acid transport proteins SLC27A1 (also called FATP1) and CD36, respectively [12, 31, 199]. Inhibition of CD36 has been found to be able to restrict the alternatively polarization of macrophages [108] and reduce the infiltration of Treg cells in tumors [200]. In addition, CD36 targeting induced additional anti-tumor responses with anti-PD-1 therapy through enhancement of anti-tumor activity in tumor-infiltrating lymphocytes [200]. These observations indicate that CD36 inhibition can provide consistent benefits through directly blocking CAAs and/or cancer cells while limiting immunosuppressive lymphocytes. Moreover, a local decrease of lactate has potential therapeutic implications. Targeting lactate transporters with the small molecule AZ3965 can reduce the level of lactate in tumors, which is expected to achieve clinical benefits [201]. Another therapeutic strategy is to suppress the conversion of pyruvate to lactate by inactivating LDHA. In a mouse model of non-small cell lung cancer (NSCLC), the inactivation of LDHA decreased the proliferation and survival of tumor cells [202], resulting in a reduction in tumorigenesis and disease regression, indicating that LDHA has an impact on tumor cell survival and can be a promising target for NSCLC treatment [202]. Likewise, targeting LDHA induced immunosurveillance has been shown to be mediated by T and NK cells in mouse tumor models [85]. Although these findings indicate that inhibiting the production and accumulation of lactate in TAME is an interesting direction for cancer treatment, how these treatments affect anti-tumor immunity of the host and synergize with current cancer immunotherapies such as immune checkpoint blockade (ICB) remains to be investigated, especially considering that both of them function at least in part by regulating the metabolism in the TAME. Finally, the directional manipulation of the ATP-adenosine axis might be a new immunotherapeutic target to inhibit the immunosuppression mediated by CAAs. Inhibiting CD39 or CD73 by small-molecule inhibitors or blocking antibody markedly reduced the inhibition of CD4+ and CD8+ T cell responses and meanwhile elevated the cytotoxic activity of CTL and NK cells, resulting in tumor cells being killed [203]. The combined treatment with these inhibitors and ICB suppressed tumor growth and induced a powerful synergistic effect on survival in several clinical and preclinical studies [203]. Targeting therapies provide opportunities to combine various blockers targeting the ATP-adenosine axis into a more comprehensive treatment approach.

The advent of ICB indicates a brand-new era of immuno-oncology and proves the feasibility to utilize the potential of the patient’s own immune system in cancer treatment [204]. Considering the possible metabolic changes caused by checkpoint blockade and the importance of metabolic reprogramming downstream of T cell signals, targeting metabolic factors during ICB treatment may produce therapeutic synergies. This view is proven in a small retrospective study that great clinical responses were achieved by the combination of metformin and checkpoint blockade [205]. Independent of ICB treatment, metformin is shown to have a beneficial impact on TILs [206] by inhibiting the secretion of cytokines (VEGF, IL-6, MMP9 and FGF2) by adipocytes [207–209], indicating that the compound might be very suitable for treatment combining with ICB. In addition, metformin influences metabolism through altering mitochondrial respiration and stimulating the energy sensor AMP-activated protein kinase (AMPK) [210]. Furthermore, AMPK-dependent phosphorylation of serine residue 195 on programmed cell death 1 ligand 1 (PD- L1) changes its glycan structure, thereby promoting its degradation [211]. In the biopsy samples of breast cancer patients, there was a significant association between AMPK activation, the decrease of PD-L1 expression level and the clinical response, indicating that this effect may also be clinically relevant [211]. The above findings indicate that metformin is a promising compound as a combination therapy with checkpoint blockade, which deserves further development.

## Conclusions

**Fig. 2:**
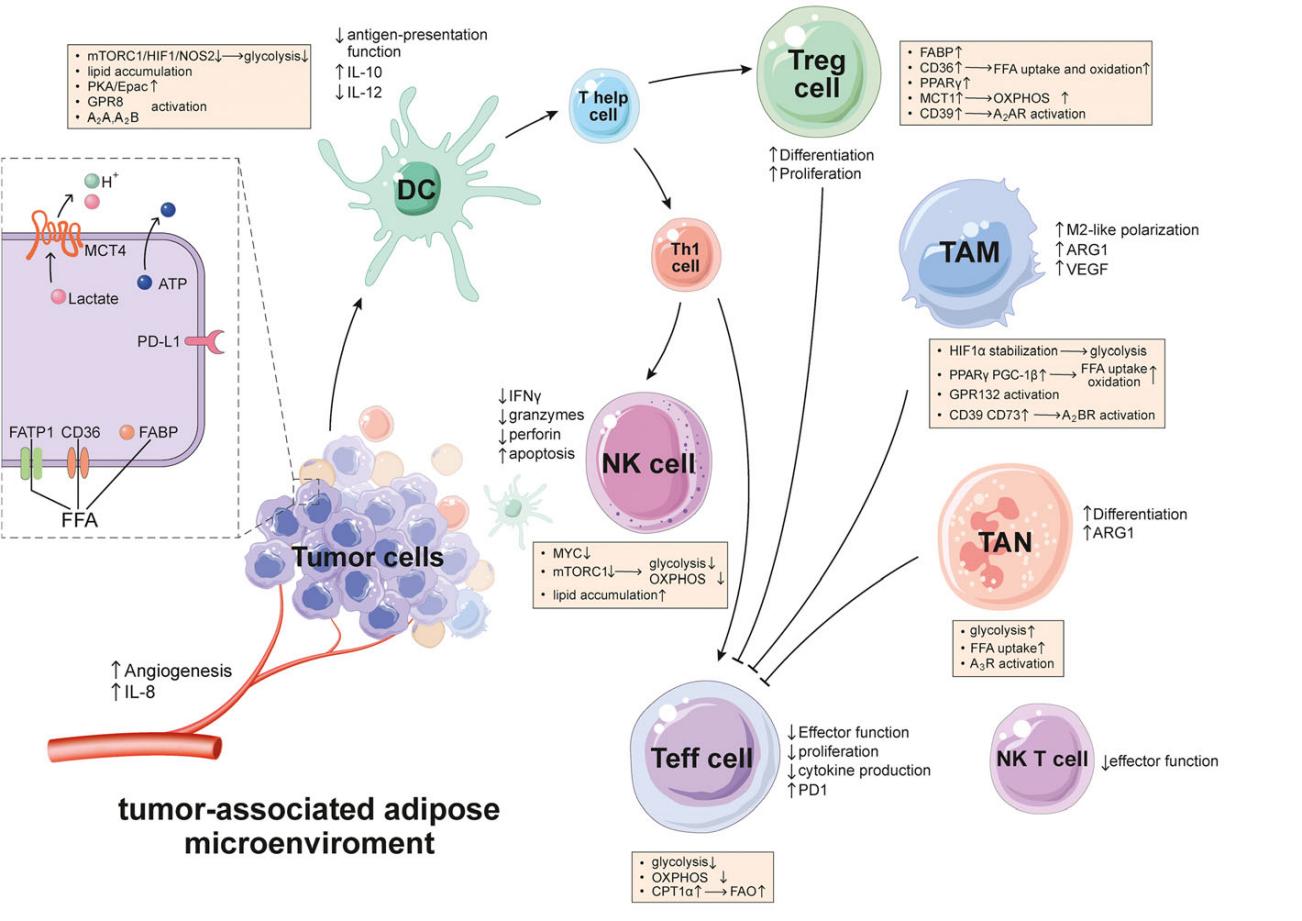
CAAs in the tumor-associated adipose microenvironment. The tumor microenvironment is composed of various types of cells, including cancer cells, stromal and immune cells. CAAs are one of the most important components that play an important role in the progression and development of cancer via metabolic reprogramming and cytokines interacting with tumor cells and immune cells, such as macrophages, T cells, NK cells and dendritic cells. CAAs inhibit the differentiation and proliferation of Teff cells and NK T cells, while play a positive effect on that of Tregs and TANs. CAAs also facilitate the alternatively polarization of macrophages to a M2-like phenotype, promoting the invasion and migration. Overall, CAAs interacts with stromal cells and immune cells to facilitate tumor progression. ARG1:arginase 1; ATP: adenosine triphosphate; CD36: cluster of differentiation 36; CPT-1α: carnitine palmitoyl transferase 1α; DC: dendritic cells; FABP: fatty acid-binding protein; FAO: fatty acid oxidation; FATP1: fatty acid transport protein 1; FFA: free fatty acid; GPR132: G protein-coupled receptor 132; GPR8: G protein-coupled receptor 8; HIF1: hypoxia inducible factor-1; IL-10: interleukin-10; IL-12: interleukin-12; MCT1: monocarboxylate transporter 1; MCT4: monocarboxylate transporter 4; mTORC1: mammalian target of rapamycin complex 1; NK cell: natural killer cell; NOS2: nitric oxide synthase 2; OXPHOS: oxidative phosphorylation; PD1: programmed cell death protein 1; PD-L1: programmed cell death 1 ligand 1; PGC-1β: peroxisome proliferator-activated receptor-γ coactivator 1β; PKA: protein kinase A; PPARγ: peroxisome proliferators-activated receptor γ; TAM: tumor-associated macrophage; TAN: tumor-associated neutrophil; Tregs: T regulatory cells; VEGF: vascular endothelial growth factor

In this context, we have highlighted how CAAs act as a major effector in the immune system (Fig. 2, Table 1). Although most cell bioenergetics findings were not based on CAAs originally, we used CAAs as an example to illustrate how tissues set up processes to control metabolic phenotypes at the cellular level. Understanding the metabolic control on the effector and the fate of leukocytes will ultimately open doors to manipulate the immune system in both metabolic disorders and other situations such as in the tumor microenvironment. In addition, learning how to regulate metabolic pathways or metabolites in immune subgroups might have an essential effect on preventing obesity-promoted tumor progression.

Increasing numbers of research studies are concentrated on CAAs. Increasing functional analysis in preclinical models and relevant analysis of patient materials suggest that targeting CAAs could improve treatment strategies. A variety of therapeutic strategies have been developed that directly target CAAs or functional mediators. In addition, many anticancer drugs previously tested in humans might also target CAAs or the modulators. For example, the inhibitors targeting the JAK1/STAT3 pathway play a role in both tumor cells and CAAs. Hence, it is worth investigating whether these inhibitors could suppress CAA production and activation, thereby eliminating CAA formation in the TME [6, 212].

To accelerate the leap from laboratory to hospital bed, there are some challenges and unmet needs for CAA therapies to overcome. First, due to the lack of analytical methods that can be used to dissect the molecular landscape of CAAs, the underlying mechanism for CAA formation in different cancer types remains elusive. Are CAAs just distinctive cellular phenotypes of the same kind of cell that have adapted to diverse TMEs in different tumor types or stages? Deep genomic sequencing is required for the rapid development of CAA-specific diagnostic or prognostic approaches, as well as CAA-targeted therapies. Considering heterogeneity, is there a subset of protumor CAAs that exhibits an antitumorigenic phenotype? How do epigenetic factors affect the gene expression patterns and biological behavior of CAAs? To excavate the potential of CAA-targeted therapies as a new anti-tumor strategy, these issues must be addressed.

Since CAAs are involved in the complex intercellular interactions in the TME, targeting CAA might induce multifaceted stromal responses in TAME that are difficult to predict and may vary with the patients. Although the details of the TAME network are still unknown, actions could be adopted to eliminate tumor-associated inflammatory and immunosuppressive cells, to mobilize immune effector cells to kill cancer cells, and to reprogram the desmoplastic matrix to improve the delivery of anti-tumor agents. Therefore, targeting CAA can not only inhibit the “seeds” of cancer but can also transform the “soil” of cancer to build a tumor-inhibiting microenvironment, thereby turning enemies that promote tumor progression into friends that inhibit tumor growth or metastasis. To finally extirpate tumors, a synergistic combination of CAA-targeted therapies and other effective therapies, including immunotherapy, should be considered.

Recently, anti-tumor therapies targeting CAAs are rapidly being investigated and developed. With the advancement of technologies, including single-cell sequencing and new biomaterials for cell-type-specific delivery, the ability to selectively eliminate or reverse the tumor-promoting CAAs can be a promising therapeutic strategy to use alone or in combination with other effective therapies for tumor treatment.

## Data Availability

Not applicable.

## Abbreviations

AMPK: AMP-activated protein kinase
APCs: Antigen-presenting cells
ARG1: Arginase 1
ATM: Adipose tissue macrophages
ATP: Adenosine triphosphate
ATs: Adipose tissues
B2M: β2-microglobulin
BMI: Body mass index
CAAs: Cancer-associated adipocytes
Cav-1: Caveolin-1
CCL2: Chemokine (C–C motif) ligand 2
CD36: Cluster of differentiation 36
CLSs: Crown-like structures
CMS: Citrate-malate shuttle
CPT-1α: Carnitine palmitoyl transferase 1α
CSF: Colony-stimulating factor
DC: Dendritic cells
ER: Endoplasmic reticulum
FABP: Fatty acid-binding protein
FAO: Fatty acid oxidation
FATP1: Fatty acid transport protein 1
FFA: Free fatty acid
GPR132: G protein-coupled receptor 132
GPR8: G protein-coupled receptor 8
HFD: High-fat diet
HIF1: Hypoxia inducible factor-1
ICB: Immune checkpoint blockade
IL-10: Interleukin-10
IL-12: Interleukin-12
LAL: Lysosomal acid lipase
LDH: Lactate dehydrogenase
LPS: Lipopolysaccharides
MCT1: Monocarboxylate transporter 1
MCT4: Monocarboxylate transporter 4
MDSCs: Myeloid-derived suppressor cells
mTORC1: Mammalian target of rapamycin complex 1
NETs: Neutrophil extracellular traps
NK cell: Natural killer cell
NO: nitrIC OXIDE
NOS2: NITRIC oxide synthase 2
NSCLC: Non-small cell lung cancer
OIS: Oncogene-induced senescence
OXPHOS: Oxidative phosphorylation
PD1: Programmed cell death protein 1
PDAC: Pancreatic ductal adenocarcinoma
PDK1: Pyruvate dehydrogenase kinase 1
PD-L1: Programmed cell death 1 ligand 1
PGC-1β: Peroxisome proliferator-activated receptor-γ coactivator 1β
PHD2: Prolyl hydroxylase 2
PKA: Protein kinase A
PKM2: Pyruvate kinase M2
PPARγ: Peroxisome proliferators-activated receptor γ
PPP: Pentose phosphate pathway
PSCs: Pancreatic stellate cells
PTHrP: Parathyroid hormone-related protein
PUFAs: Polyunsaturated fatty acids
RCCs: Renal cell carcinomas
ROS: Reactive oxygen species
SASP: Senescence-associated secretory phenotype
SIRT1: Silent information regulator 2 homolog 1
TAGs: Triacylglycerols
TAM: Tumor-associated macrophage
TAME: Tumor-adipose microenvironment
TAN: Tumor-associated neutrophil
TNF-α: Tumor necrosis factor-α
Tregs: T regulatory cells
VEGF: Vascular endothelial growth factor

## References

[1] Calle EE, Rodriguez C, Walker-Thurmond K, Thun MJ (2003). Overweight, obesity, and mortality from cancer in a prospectively studied cohort of U.S. adults. N Engl J Med.

[2] Calle EE, Kaaks R (2004). Overweight, obesity and cancer: epidemiological evidence and proposed mechanisms. Nat Rev Cancer.

[3] Picon-Ruiz M, Morata-Tarifa C, Valle-Goffin JJ, Friedman ER, Slingerland JM (2017). Obesity and adverse breast cancer risk and outcome: mechanistic insights and strategies for intervention. CA Cancer J Clin.

[4] Wu Q, Li B, Sun S, Sun S (2020). Unraveling adipocytes and Cancer links: is there a role for senescence?. Front Cell Dev Biol.

[5] Wu Q, Li B, Li Z, Li J, Sun S, Sun S (2019). Cancer-associated adipocytes: key players in breast cancer progression. J Hematol Oncol.

[6] Lapeire L, Hendrix A, Lambein K, Van Bockstal M, Braems G, Van Den Broecke R, Limame R, Mestdagh P, Vandesompele J, Vanhove C (2014). Cancer-associated adipose tissue promotes breast cancer progression by paracrine oncostatin M and Jak/STAT3 signaling. Cancer Res.

[7] Lazar I, Clement E, Dauvillier S, Milhas D, Ducoux-Petit M, LeGonidec S, Moro C, Soldan V, Dalle S, Balor S (2016). Adipocyte Exosomes promote melanoma aggressiveness through fatty acid oxidation: a novel mechanism linking obesity and Cancer. Cancer Res.

[8] Kohlgruber AC, LaMarche NM, Lynch L (2016). Adipose tissue at the nexus of systemic and cellular immunometabolism. Semin Immunol.

[9] Dirat B, Bochet L, Dabek M, Daviaud D, Dauvillier S, Majed B, Wang YY, Meulle A, Salles B, Le Gonidec S (2011). Cancer-associated adipocytes exhibit an activated phenotype and contribute to breast cancer invasion. Cancer Res.

[10] Deng T, Lyon CJ, Bergin S, Caligiuri MA, Hsueh WA (2016). Obesity, inflammation, and Cancer. Annu Rev Pathol.

[11] Iyengar P, Espina V, Williams TW, Lin Y, Berry D, Jelicks LA, Lee H, Temple K, Graves R, Pollard J (2005). Adipocyte-derived collagen VI affects early mammary tumor progression in vivo, demonstrating a critical interaction in the tumor/stroma microenvironment. J Clin Invest.

[12] Wu Q, Li J, Li Z, Sun S, Zhu S, Wang L, Wu J, Yuan J, Zhang Y, Sun S (2019). Exosomes from the tumour-adipocyte interplay stimulate beige/brown differentiation and reprogram metabolism in stromal adipocytes to promote tumour progression. J Exp Clin Cancer Res.

[13] Singh R, Parveen M, Basgen JM, Fazel S, Meshesha MF, Thames EC, Moore B, Martinez L, Howard CB, Vergnes L (2016). Increased expression of beige/Brown adipose markers from host and breast Cancer cells influence Xenograft formation in mice. Mol Cancer Res.

[14] Tchkonia T, Morbeck DE, Von Zglinicki T, Van Deursen J, Lustgarten J, Scrable H, Khosla S, Jensen MD, Kirkland JL (2010). Fat tissue, aging, and cellular senescence. Aging Cell.

[15] Ghosh AK, O'Brien M, Mau T, Qi N, Yung R (2019). Adipose tissue senescence and inflammation in aging is reversed by the young milieu. J Gerontol A Biol Sci Med Sci.

[16] Ishay-Ronen D, Diepenbruck M, Kalathur RKR, Sugiyama N, Tiede S, Ivanek R, Bantug G, Morini MF, Wang J, Hess C (2019). Gain fat-lose metastasis: converting invasive breast Cancer cells into adipocytes inhibits Cancer metastasis. Cancer Cell.

[17] Sanchez-Vega F, Mina M, Armenia J, Chatila WK, Luna A, La KC, Dimitriadoy S, Liu DL, Kantheti HS, Saghafinia S (2018). Oncogenic Signaling Pathways in The Cancer Genome Atlas. Cell.

[18] Fairfield H, Dudakovic A, Khatib CM, Farrell M, Costa S, Falank C, Hinge M, Murphy CS, DeMambro V, Pettitt JA, et al. Myeloma-modified adipocytes exhibit metabolic dysfunction and a senescence-associated secretory phenotype (SASP). Cancer Res. 2020. 10.1158/0008-5472.CAN-20-1088 PMC7854508 33218968

[19] Wang S, Wang N, Zheng Y, Zhang J, Zhang F, Wang Z (2017). Caveolin-1: An oxidative stress-related target for Cancer prevention. Oxidative Med Cell Longev.

[20] Witkiewicz AK, Dasgupta A, Sotgia F, Mercier I, Pestell RG, Sabel M, Kleer CG, Brody JR, Lisanti MP (2009). An absence of stromal caveolin-1 expression predicts early tumor recurrence and poor clinical outcome in human breast cancers. Am J Pathol.

[21] Martinez-Outschoorn UE, Trimmer C, Lin Z, Whitaker-Menezes D, Chiavarina B, Zhou J, Wang C, Pavlides S, Martinez-Cantarin MP, Capozza F (2010). Autophagy in cancer associated fibroblasts promotes tumor cell survival: role of hypoxia, HIF1 induction and NFkappaB activation in the tumor stromal microenvironment. Cell Cycle.

[22] Yu DM, Jung SH, An HT, Lee S, Hong J, Park JS, Lee H, Lee H, Bahn MS, Lee HC (2017). Caveolin-1 deficiency induces premature senescence with mitochondrial dysfunction. Aging Cell.

[23] Burd CE, Sorrentino JA, Clark KS, Darr DB, Krishnamurthy J, Deal AM, Bardeesy N, Castrillon DH, Beach DH, Sharpless NE (2013). Monitoring tumorigenesis and senescence in vivo with a p16(INK4a)-luciferase model. Cell.

[24] Nelson G, Wordsworth J, Wang C, Jurk D, Lawless C, Martin-Ruiz C, von Zglinicki T (2012). A senescent cell bystander effect: senescence-induced senescence. Aging Cell.

[25] Acosta JC, Banito A, Wuestefeld T, Georgilis A, Janich P, Morton JP, Athineos D, Kang TW, Lasitschka F, Andrulis M (2013). A complex secretory program orchestrated by the inflammasome controls paracrine senescence. Nat Cell Biol.

[26] Acosta JC, O'Loghlen A, Banito A, Guijarro MV, Augert A, Raguz S, Fumagalli M, Da Costa M, Brown C, Popov N (2008). Chemokine signaling via the CXCR2 receptor reinforces senescence. Cell.

[27] Nelson G, Kucheryavenko O, Wordsworth J, von Zglinicki T (2018). The senescent bystander effect is caused by ROS-activated NF-kappaB signalling. Mech Ageing Dev.

[28] Yang H, Wang H, Ren J, Chen Q, Chen ZJ (2017). cGAS is essential for cellular senescence. Proc Natl Acad Sci U S A.

[29] Petruzzelli M, Schweiger M, Schreiber R, Campos-Olivas R, Tsoli M, Allen J, Swarbrick M, Rose-John S, Rincon M, Robertson G (2014). A switch from white to brown fat increases energy expenditure in cancer-associated cachexia. Cell Metab.

[30] Kir S, White JP, Kleiner S, Kazak L, Cohen P, Baracos VE, Spiegelman BM (2014). Tumour-derived PTH-related protein triggers adipose tissue browning and cancer cachexia. Nature.

[31] Wu Q, Sun S, Li Z, Yang Q, Li B, Zhu S, Wang L, Wu J, Yuan J, Wang C (2019). Breast cancer-released exosomes trigger cancer-associated cachexia to promote tumor progression. Adipocyte.

[32] Wu Q, Sun S, Li Z, Yang Q, Li B, Zhu S, Wang L, Wu J, Yuan J, Yang C (2018). Tumour-originated exosomal miR-155 triggers cancer-associated cachexia to promote tumour progression. Mol Cancer.

[33] Wang YY, Attane C, Milhas D, Dirat B, Dauvillier S, Guerard A, Gilhodes J, Lazar I, Alet N, Laurent V (2017). Mammary adipocytes stimulate breast cancer invasion through metabolic remodeling of tumor cells. JCI Insight.

[34] Kratz M, Coats BR, Hisert KB, Hagman D, Mutskov V, Peris E, Schoenfelt KQ, Kuzma JN, Larson I, Billing PS (2014). Metabolic dysfunction drives a mechanistically distinct proinflammatory phenotype in adipose tissue macrophages. Cell Metab.

[35] Salimian Rizi B, Caneba C, Nowicka A, Nabiyar AW, Liu X, Chen K, Klopp A, Nagrath D (2015). Nitric oxide mediates metabolic coupling of omentum-derived adipose stroma to ovarian and endometrial cancer cells. Cancer Res.

[36] Sanchez-Alvarez R, Martinez-Outschoorn UE, Lamb R, Hulit J, Howell A, Gandara R, Sartini M, Rubin E, Lisanti MP, Sotgia F (2013). Mitochondrial dysfunction in breast cancer cells prevents tumor growth: understanding chemoprevention with metformin. Cell Cycle.

[37] Tozzi M, Hansen JB, Novak I (2020). Pannexin-1 mediated ATP release in adipocytes is sensitive to glucose and insulin and modulates lipolysis and macrophage migration. Acta Physiol (Oxf).

[38] Kepp O, Loos F, Liu P, Kroemer G (2017). Extracellular nucleosides and nucleotides as immunomodulators. Immunol Rev.

[39] Clement E, Lazar I, Attane C, Carrie L, Dauvillier S, Ducoux-Petit M, Esteve D, Menneteau T, Moutahir M, Le Gonidec S, et al. Adipocyte extracellular vesicles carry enzymes and fatty acids that stimulate mitochondrial metabolism and remodeling in tumor cells. EMBO J. 2020:e102525. 10.15252/embj.2019102525 PMC6996584 31919869

[40] Wang S, Xu M, Li X, Su X, Xiao X, Keating A, Zhao RC (2018). Exosomes released by hepatocarcinoma cells endow adipocytes with tumor-promoting properties. J Hematol Oncol.

[41] Zewdu A, Casadei L, Pollock RE, Braggio D (2020). Adipose Tumor Microenvironment. Adv Exp Med Biol.

[42] Bei L, Qi W, Qian Y, Zhiyu L, Juanjuan L, Juan W, Shan Z, Lijun W, Shichong L, Si S, et al. Macrophages in tumor-associated adipose microenvironment accelerate tumor progression. Research Square. 2020.

[43] Naylor C, Petri WA (2016). Leptin regulation of immune responses. Trends Mol Med.

[44] Ingram JR, Dougan M, Rashidian M, Knoll M, Keliher EJ, Garrett S, Garforth S, Blomberg OS, Espinosa C, Bhan A (2017). PD-L1 is an activation-independent marker of brown adipocytes. Nat Commun.

[45] Wu B, Sun X, Gupta HB, Yuan B, Li J, Ge F, Chiang HC, Zhang X, Zhang C, Zhang D (2018). Adipose PD-L1 modulates PD-1/PD-L1 checkpoint blockade immunotherapy efficacy in breast Cancer. Oncoimmunology.

[46] Shaul ME, Fridlender ZG (2019). Tumour-associated neutrophils in patients with cancer. Nat Rev Clin Oncol.

[47] Lee BC, Lee J (2014). Cellular and molecular players in adipose tissue inflammation in the development of obesity-induced insulin resistance. Biochim Biophys Acta.

[48] Talukdar S, Oh DY, Bandyopadhyay G, Li D, Xu J, McNelis J, Lu M, Li P, Yan Q, Zhu Y (2012). Neutrophils mediate insulin resistance in mice fed a high-fat diet through secreted elastase. Nat Med.

[49] Elgazar-Carmon V, Rudich A, Hadad N, Levy R (2008). Neutrophils transiently infiltrate intra-abdominal fat early in the course of high-fat feeding. J Lipid Res.

[50] Jensen HK, Donskov F, Marcussen N, Nordsmark M, Lundbeck F, von der Maase H (2009). Presence of intratumoral neutrophils is an independent prognostic factor in localized renal cell carcinoma. J Clin Oncol.

[51] Quail DF, Olson OC, Bhardwaj P, Walsh LA, Akkari L, Quick ML, Chen IC, Wendel N, Ben-Chetrit N, Walker J, et al. Obesity alters the lung myeloid cell landscape to enhance breast cancer metastasis through IL5 and GM-CSF. Nat Cell Biol. 2017. 10.1038/ncb3578 PMC6759922 28737771

[52] Incio J, Liu H, Suboj P, Chin SM, Chen IX, Pinter M, Ng MR, Nia HT, Grahovac J, Kao S (2016). Obesity-induced inflammation and Desmoplasia promote pancreatic Cancer progression and resistance to chemotherapy. Cancer Discov.

[53] Vazquez Rodriguez G, Abrahamsson A, Jensen LDE, Dabrosin C (2018). Adipocytes promote early steps of breast Cancer cell dissemination via Interleukin-8. Front Immunol.

[54] Rotondo R, Barisione G, Mastracci L, Grossi F, Orengo AM, Costa R, Truini M, Fabbi M, Ferrini S, Barbieri O (2009). IL-8 induces exocytosis of arginase 1 by neutrophil polymorphonuclears in nonsmall cell lung cancer. Int J Cancer.

[55] Coffelt SB, Kersten K, Doornebal CW, Weiden J, Vrijland K, Hau CS, Verstegen NJM, Ciampricotti M, Hawinkels L, Jonkers J (2015). IL-17-producing gammadelta T cells and neutrophils conspire to promote breast cancer metastasis. Nature.

[56] Rodriguez-Espinosa O, Rojas-Espinosa O, Moreno-Altamirano MM, Lopez-Villegas EO, Sanchez-Garcia FJ (2015). Metabolic requirements for neutrophil extracellular traps formation. Immunology.

[57] Sadiku P, Willson JA, Dickinson RS, Murphy F, Harris AJ, Lewis A, Sammut D, Mirchandani AS, Ryan E, Watts ER (2017). Prolyl hydroxylase 2 inactivation enhances glycogen storage and promotes excessive neutrophilic responses. J Clin Invest.

[58] Hsu BE, Tabaries S, Johnson RM, Andrzejewski S, Senecal J, Lehuede C, Annis MG, Ma EH, Vols S, Ramsay L (2019). Immature low-density neutrophils exhibit metabolic flexibility that facilitates breast Cancer liver metastasis. Cell Rep.

[59] Riffelmacher T, Clarke A, Richter FC, Stranks A, Pandey S, Danielli S, Hublitz P, Yu Z, Johnson E, Schwerd T (2017). Autophagy-dependent generation of free fatty acids is critical for Normal neutrophil differentiation. Immunity.

[60] Khalafalla FG, Greene S, Khan H, Ilves K, Monsanto MM, Alvarez R, Chavarria M, Nguyen J, Norman B, Dembitsky WP (2017). P2Y2 nucleotide receptor prompts human cardiac progenitor cell activation by modulating hippo signaling. Circ Res.

[61] Pitt JM, Kroemer G, Zitvogel L (2017). Immunogenic and non-immunogenic cell death in the tumor microenvironment. Adv Exp Med Biol.

[62] Cekic C, Linden J (2016). Purinergic regulation of the immune system. Nat Rev Immunol.

[63] Sakaki H, Tsukimoto M, Harada H, Moriyama Y, Kojima S (2013). Autocrine regulation of macrophage activation via exocytosis of ATP and activation of P2Y11 receptor. PLoS One.

[64] Stout CE, Costantin JL, Naus CC, Charles AC (2002). Intercellular calcium signaling in astrocytes via ATP release through connexin hemichannels. J Biol Chem.

[65] Bao L, Locovei S, Dahl G (2004). Pannexin membrane channels are mechanosensitive conduits for ATP. FEBS Lett.

[66] Lazarowski ER, Sesma JI, Seminario-Vidal L, Kreda SM (2011). Molecular mechanisms of purine and pyrimidine nucleotide release. Adv Pharmacol.

[67] Di Virgilio F, Sarti AC, Falzoni S, De Marchi E, Adinolfi E. Extracellular ATP and P2 purinergic signalling in the tumour microenvironment. Nat Rev Cancer. 2018. 10.1038/s41568-018-0037-0 30006588

[68] Chen Y, Corriden R, Inoue Y, Yip L, Hashiguchi N, Zinkernagel A, Nizet V, Insel PA, Junger WG (2006). ATP release guides neutrophil chemotaxis via P2Y2 and A3 receptors. Science.

[69] Cekic C, Day YJ, Sag D, Linden J (2014). Myeloid expression of adenosine A2A receptor suppresses T and NK cell responses in the solid tumor microenvironment. Cancer Res.

[70] Sullivan GW, Rieger JM, Scheld WM, Macdonald TL, Linden J (2001). Cyclic AMP-dependent inhibition of human neutrophil oxidative activity by substituted 2-propynylcyclohexyl adenosine a(2A) receptor agonists. Br J Pharmacol.

[71] Sullivan GW, Lee DD, Ross WG, DiVietro JA, Lappas CM, Lawrence MB, Linden J (2004). Activation of A2A adenosine receptors inhibits expression of alpha 4/beta 1 integrin (very late antigen-4) on stimulated human neutrophils. J Leukoc Biol.

[72] Keppel MP, Saucier N, Mah AY, Vogel TP, Cooper MA (2015). Activation-specific metabolic requirements for NK cell IFN-gamma production. J Immunol.

[73] Marçais A, Cherfils-Vicini J, Viant C, Degouve S, Viel S, Fenis A, Rabilloud J, Mayol K, Tavares A, Bienvenu J (2014). The metabolic checkpoint kinase mTOR is essential for IL-15 signaling during the development and activation of NK cells. Nat Immunol.

[74] Keppel MP, Saucier N, Mah AY, Vogel TP, Cooper MA (2015). Activation-specific metabolic requirements for NK cell IFN-γ production. J Immunol.

[75] Donnelly RP, Loftus RM, Keating SE, Liou KT, Biron CA, Gardiner CM, Finlay DK (2014). mTORC1-dependent metabolic reprogramming is a prerequisite for NK cell effector function. J Immunol.

[76] Assmann N, O'Brien KL, Donnelly RP, Dyck L, Zaiatz-Bittencourt V, Loftus RM, Heinrich P, Oefner PJ, Lynch L, Gardiner CM (2017). Srebp-controlled glucose metabolism is essential for NK cell functional responses. Nat Immunol.

[77] Michelet X, Dyck L, Hogan A, Loftus RM, Duquette D, Wei K, Beyaz S, Tavakkoli A, Foley C, Donnelly R (2018). Metabolic reprogramming of natural killer cells in obesity limits antitumor responses. Nat Immunol.

[78] Schafer JR, Salzillo TC, Chakravarti N, Kararoudi MN, Trikha P, Foltz JA, Wang R, Li S, Lee DA (2019). Education-dependent activation of glycolysis promotes the cytolytic potency of licensed human natural killer cells. J Allergy Clin Immunol.

[79] O'Rourke RW, Metcalf MD, White AE, Madala A, Winters BR, Maizlin II, Jobe BA, Roberts CT, Slifka MK, Marks DL (2009). Depot-specific differences in inflammatory mediators and a role for NK cells and IFN-gamma in inflammation in human adipose tissue. Int J Obes.

[80] O'Rourke RW, Meyer KA, Neeley CK, Gaston GD, Sekhri P, Szumowski M, Zamarron B, Lumeng CN, Marks DL (2014). Systemic NK cell ablation attenuates intra-abdominal adipose tissue macrophage infiltration in murine obesity. Obesity (Silver Spring).

[81] Wensveen FM, Jelencic V, Valentic S, Sestan M, Wensveen TT, Theurich S, Glasner A, Mendrila D, Stimac D, Wunderlich FT (2015). NK cells link obesity-induced adipose stress to inflammation and insulin resistance. Nat Immunol.

[82] Xu L, Shen M, Chen X, Zhu R, Yang DR, Tsai Y, Keng PC, Chen Y, Lee SO (2018). Adipocytes affect castration-resistant prostate cancer cells to develop the resistance to cytotoxic action of NK cells with alterations of PD-L1/NKG2D ligand levels in tumor cells. Prostate.

[83] Tobin LM, Mavinkurve M, Carolan E, Kinlen D, O'Brien EC, Little MA, Finlay DK, Cody D, Hogan AE, O'Shea D (2017). NK cells in childhood obesity are activated, metabolically stressed, and functionally deficient. JCI insight.

[84] Abarca-Rojano E, Muniz-Hernandez S, Moreno-Altamirano MM, Mondragon-Flores R, Enriquez-Rincon F, Sanchez-Garcia FJ (2009). Re-organization of mitochondria at the NK cell immune synapse. Immunol Lett.

[85] Brand A, Singer K, Koehl GE, Kolitzus M, Schoenhammer G, Thiel A, Matos C, Bruss C, Klobuch S, Peter K (2016). LDHA-associated lactic acid production blunts tumor Immunosurveillance by T and NK cells. Cell Metab.

[86] Harmon C, Robinson MW, Hand F, Almuaili D, Mentor K, Houlihan DD, Hoti E, Lynch L, Geoghegan J, O'Farrelly C (2019). Lactate-mediated acidification of tumor microenvironment induces apoptosis of liver-resident NK cells in colorectal liver metastasis. Cancer immunology research.

[87] Souza-Fonseca-Guimaraes F, Cursons J, Huntington ND (2019). The emergence of natural killer cells as a major target in Cancer immunotherapy. Trends Immunol.

[88] Pei B, Vela JL, Zajonc D, Kronenberg M (2012). Interplay between carbohydrate and lipid in recognition of glycolipid antigens by natural killer T cells. Ann N Y Acad Sci.

[89] De Libero G, Mori L (2005). Recognition of lipid antigens by T cells. Nat Rev Immunol.

[90] Zeng X, Zhou J, Xiong Z, Sun H, Yang W, Mok MTS, Wang J, Li J, Liu M, Tang W, et al. Cell cycle-related kinase reprograms the liver immune microenvironment to promote cancer metastasis. Cell Mol Immunol. 2020. 10.1038/s41423-020-00534-2 PMC8115036 32879468

[91] Ma C, Han M, Heinrich B, Fu Q, Zhang Q, Sandhu M, Agdashian D, Terabe M, Berzofsky JA, Fako V (2018). Gut microbiome-mediated bile acid metabolism regulates liver cancer via NKT cells. Science (New York, NY).

[92] Ji Y, Sun S, Xu A, Bhargava P, Yang L, Lam KS, Gao B, Lee CH, Kersten S, Qi L (2012). Activation of natural killer T cells promotes M2 macrophage polarization in adipose tissue and improves systemic glucose tolerance via interleukin-4 (IL-4)/STAT6 protein signaling axis in obesity. J Biol Chem.

[93] Lynch L, Nowak M, Varghese B, Clark J, Hogan AE, Toxavidis V, Balk SP, O'Shea D, O'Farrelly C, Exley MA (2012). Adipose tissue invariant NKT cells protect against diet-induced obesity and metabolic disorder through regulatory cytokine production. Immunity.

[94] Ohmura K, Ishimori N, Ohmura Y, Tokuhara S, Nozawa A, Horii S, Andoh Y, Fujii S, Iwabuchi K, Onoe K (2010). Natural killer T cells are involved in adipose tissues inflammation and glucose intolerance in diet-induced obese mice. Arterioscler Thromb Vasc Biol.

[95] Cox N, Geissmann F (2019). Macrophage ontogeny in the control of adipose tissue biology. Curr Opin Immunol.

[96] Chylikova J, Dvorackova J, Tauber Z, Kamarad V (2018). M1/M2 macrophage polarization in human obese adipose tissue. Biomedical papers of the Medical Faculty of the University Palacky, Olomouc, Czechoslovakia.

[97] Biswas SK, Mantovani A (2010). Macrophage plasticity and interaction with lymphocyte subsets: cancer as a paradigm. Nat Immunol.

[98] Perdiguero EG, Geissmann F (2016). The development and maintenance of resident macrophages. Nat Immunol.

[99] Howe LR, Subbaramaiah K, Hudis CA, Dannenberg AJ (2013). Molecular pathways: adipose inflammation as a mediator of obesity-associated cancer. Clin Cancer Res.

[100] Cha YJ, Kim ES, Koo JS. Tumor-associated macrophages and crown-like structures in adipose tissue in breast cancer. Breast cancer research and treatment. 2018. 10.1007/s10549-018-4722-1 29468486

[101] Engin AB, Engin A, Gonul II (2019). The effect of adipocyte-macrophage crosstalk in obesity-related breast cancer. J Mol Endocrinol.

[102] Shaul ME, Bennett G, Strissel KJ, Greenberg AS, Obin MS (2010). Dynamic, M2-like remodeling phenotypes of CD11c+ adipose tissue macrophages during high-fat diet--induced obesity in mice. Diabetes.

[103] Van den Bossche J, Baardman J, de Winther MP. Metabolic characterization of polarized M1 and M2 bone marrow-derived macrophages using real-time extracellular flux analysis. J Visualized Experiments. 2015;105. 10.3791/53424 PMC4692751 26649578

[104] Palsson-McDermott EM, Curtis AM, Goel G, Lauterbach MA, Sheedy FJ, Gleeson LE, van den Bosch MW, Quinn SR, Domingo-Fernandez R, Johnston DG (2015). Pyruvate kinase M2 regulates Hif-1alpha activity and IL-1beta induction and is a critical determinant of the Warburg effect in LPS-activated macrophages. Cell Metab.

[105] Suzuki H, Hisamatsu T, Chiba S, Mori K, Kitazume MT, Shimamura K, Nakamoto N, Matsuoka K, Ebinuma H, Naganuma M (2016). Glycolytic pathway affects differentiation of human monocytes to regulatory macrophages. Immunol Lett.

[106] Chiba S, Hisamatsu T, Suzuki H, Mori K, Kitazume MT, Shimamura K, Mizuno S, Nakamoto N, Matsuoka K, Naganuma M (2017). Glycolysis regulates LPS-induced cytokine production in M2 polarized human macrophages. Immunol Lett.

[107] Chen DP, Ning WR, Jiang ZZ, Peng ZP, Zhu LY, Zhuang SM, Kuang DM, Zheng L, Wu Y (2019). Glycolytic activation of peritumoral monocytes fosters immune privilege via the PFKFB3-PD-L1 axis in human hepatocellular carcinoma. J Hepatol.

[108] Huang SC, Everts B, Ivanova Y, O'Sullivan D, Nascimento M, Smith AM, Beatty W, Love-Gregory L, Lam WY, O'Neill CM (2014). Cell-intrinsic lysosomal lipolysis is essential for alternative activation of macrophages. Nat Immunol.

[109] Odegaard JI, Ricardo-Gonzalez RR, Goforth MH, Morel CR, Subramanian V, Mukundan L, Red Eagle A, Vats D, Brombacher F, Ferrante AW (2007). Macrophage-specific PPARgamma controls alternative activation and improves insulin resistance. Nature.

[110] Niu Z, Shi Q, Zhang W, Shu Y, Yang N, Chen B, Wang Q, Zhao X, Chen J, Cheng N (2017). Caspase-1 cleaves PPARgamma for potentiating the pro-tumor action of TAMs. Nat Commun.

[111] Vats D, Mukundan L, Odegaard JI, Zhang L, Smith KL, Morel CR, Wagner RA, Greaves DR, Murray PJ, Chawla A (2006). Oxidative metabolism and PGC-1beta attenuate macrophage-mediated inflammation. Cell Metab.

[112] Osborn O, Olefsky JM (2012). The cellular and signaling networks linking the immune system and metabolism in disease. Nat Med.

[113] Bourlier V, Zakaroff-Girard A, Miranville A, De Barros S, Maumus M, Sengenes C, Galitzky J, Lafontan M, Karpe F, Frayn KN (2008). Remodeling phenotype of human subcutaneous adipose tissue macrophages. Circulation.

[114] Mayi TH, Daoudi M, Derudas B, Gross B, Bories G, Wouters K, Brozek J, Caiazzo R, Raverdi V, Pigeyre M (2012). Human adipose tissue macrophages display activation of cancer-related pathways. J Biol Chem.

[115] Montalban Del Barrio I, Penski C, Schlahsa L, Stein RG, Diessner J, Wockel A, Dietl J, Lutz MB, Mittelbronn M, Wischhusen J (2016). Adenosine-generating ovarian cancer cells attract myeloid cells which differentiate into adenosine-generating tumor associated macrophages - a self-amplifying, CD39- and CD73-dependent mechanism for tumor immune escape. Journal for immunotherapy of cancer.

[116] Colegio OR, Chu NQ, Szabo AL, Chu T, Rhebergen AM, Jairam V, Cyrus N, Brokowski CE, Eisenbarth SC, Phillips GM (2014). Functional polarization of tumour-associated macrophages by tumour-derived lactic acid. Nature.

[117] Chen P, Zuo H, Xiong H, Kolar MJ, Chu Q, Saghatelian A, Siegwart DJ, Wan Y (2017). Gpr132 sensing of lactate mediates tumor-macrophage interplay to promote breast cancer metastasis. Proc Natl Acad Sci U S A.

[118] Rodriguez GM, Galpin KJC, McCloskey CW, Vanderhyden BC (2018). The Tumor Microenvironment of Epithelial Ovarian Cancer and Its Influence on Response to Immunotherapy. Cancers (Basel).

[119] Ostrand-Rosenberg S (2018). Myeloid derived-suppressor cells: their role in cancer and obesity. Curr Opin Immunol.

[120] Clements VK, Long T, Long R, Figley C, Smith DMC, Ostrand-Rosenberg S (2018). Frontline science: high fat diet and leptin promote tumor progression by inducing myeloid-derived suppressor cells. J Leukoc Biol.

[121] Yan D, Yang Q, Shi M, Zhong L, Wu C, Meng T, Yin H, Zhou J (2013). Polyunsaturated fatty acids promote the expansion of myeloid-derived suppressor cells by activating the JAK/STAT3 pathway. Eur J Immunol.

[122] Al-Khami AA, Zheng L, Del Valle L, Hossain F, Wyczechowska D, Zabaleta J, Sanchez MD, Dean MJ, Rodriguez PC, Ochoa AC (2017). Exogenous lipid uptake induces metabolic and functional reprogramming of tumor-associated myeloid-derived suppressor cells. Oncoimmunology.

[123] Steinman RM (2008). Dendritic cells in vivo: a key target for a new vaccine science. Immunity.

[124] Gardner A, Ruffell B. Dendritic cells and Cancer immunity. Trends Immunol. 2016. 10.1016/j.it.2016.09.006 PMC5135568 27793569

[125] Merad M, Sathe P, Helft J, Miller J, Mortha A (2013). The dendritic cell lineage: ontogeny and function of dendritic cells and their subsets in the steady state and the inflamed setting. Annu Rev Immunol.

[126] Le Mercier I, Poujol D, Sanlaville A, Sisirak V, Gobert M, Durand I, Dubois B, Treilleux I, Marvel J, Vlach J (2013). Tumor promotion by intratumoral plasmacytoid dendritic cells is reversed by TLR7 ligand treatment. Cancer Res.

[127] Joffre OP, Segura E, Savina A, Amigorena S (2012). Cross-presentation by dendritic cells. Nat Rev Immunol.

[128] Spranger S, Dai D, Horton B, Gajewski TF (2017). Tumor-residing Batf3 dendritic cells are required for effector T cell trafficking and adoptive T cell therapy. Cancer Cell.

[129] Laoui D, Keirsse J, Morias Y, Van Overmeire E, Geeraerts X, Elkrim Y, Kiss M, Bolli E, Lahmar Q, Sichien D (2016). The tumour microenvironment harbours ontogenically distinct dendritic cell populations with opposing effects on tumour immunity. Nat Commun.

[130] Bottcher JP, Bonavita E, Chakravarty P, Blees H, Cabeza-Cabrerizo M, Sammicheli S, Rogers NC, Sahai E, Zelenay S, Reis ESC (2018). NK cells stimulate recruitment of cDC1 into the tumor microenvironment promoting Cancer immune control. Cell.

[131] Bertola A, Ciucci T, Rousseau D, Bourlier V, Duffaut C, Bonnafous S, Blin-Wakkach C, Anty R, Iannelli A, Gugenheim J (2012). Identification of adipose tissue dendritic cells correlated with obesity-associated insulin-resistance and inducing Th17 responses in mice and patients. Diabetes.

[132] Stefanovic-Racic M, Yang X, Turner MS, Mantell BS, Stolz DB, Sumpter TL, Sipula IJ, Dedousis N, Scott DK, Morel PA (2012). Dendritic cells promote macrophage infiltration and comprise a substantial proportion of obesity-associated increases in CD11c+ cells in adipose tissue and liver. Diabetes.

[133] Jaensson E, Uronen-Hansson H, Pabst O, Eksteen B, Tian J, Coombes JL, Berg PL, Davidsson T, Powrie F, Johansson-Lindbom B (2008). Small intestinal CD103+ dendritic cells display unique functional properties that are conserved between mice and humans. J Exp Med.

[134] Everts B, Amiel E, Huang SC, Smith AM, Chang CH, Lam WY, Redmann V, Freitas TC, Blagih J, van der Windt GJ (2014). TLR-driven early glycolytic reprogramming via the kinases TBK1-IKKvarepsilon supports the anabolic demands of dendritic cell activation. Nat Immunol.

[135] Lawless SJ, Kedia-Mehta N, Walls JF, McGarrigle R, Convery O, Sinclair LV, Navarro MN, Murray J, Finlay DK (2017). Glucose represses dendritic cell-induced T cell responses. Nat Commun.

[136] Wu D, Sanin DE, Everts B, Chen Q, Qiu J, Buck MD, Patterson A, Smith AM, Chang CH, Liu Z (2016). Type 1 Interferons induce changes in Core metabolism that are critical for immune function. Immunity.

[137] Herber DL, Cao W, Nefedova Y, Novitskiy SV, Nagaraj S, Tyurin VA, Corzo A, Cho HI, Celis E, Lennox B (2010). Lipid accumulation and dendritic cell dysfunction in cancer. Nat Med.

[138] Li L, Huang L, Ye H, Song SP, Bajwa A, Lee SJ, Moser EK, Jaworska K, Kinsey GR, Day YJ (2012). Dendritic cells tolerized with adenosine a(2)AR agonist attenuate acute kidney injury. J Clin Invest.

[139] Nasi A, Fekete T, Krishnamurthy A, Snowden S, Rajnavolgyi E, Catrina AI, Wheelock CE, Vivar N, Rethi B (2013). Dendritic cell reprogramming by endogenously produced lactic acid. J Immunol.

[140] Kayhan M, Koyas A, Akdemir I, Savas AC, Cekic C (2019). Adenosine receptor signaling targets both PKA and Epac pathways to polarize dendritic cells to a suppressive phenotype. J Immunol.

[141] Caronni N, Simoncello F, Stafetta F, Guarnaccia C, Ruiz-Moreno JS, Opitz B, Galli T, Proux-Gillardeaux V, Benvenuti F (2018). Downregulation of membrane trafficking proteins and lactate conditioning determine loss of dendritic cell function in lung Cancer. Cancer Res.

[142] Ranganathan P, Shanmugam A, Swafford D, Suryawanshi A, Bhattacharjee P, Hussein MS, Koni PA, Prasad PD, Kurago ZB, Thangaraju M (2018). GPR81, a cell-surface receptor for lactate, regulates intestinal homeostasis and protects mice from experimental colitis. J Immunol.

[143] Fontenot JD, Gavin MA, Rudensky AY (2003). Foxp3 programs the development and function of CD4+CD25+ regulatory T cells. Nat Immunol.

[144] Abbas AK, Benoist C, Bluestone JA, Campbell DJ, Ghosh S, Hori S, Jiang S, Kuchroo VK, Mathis D, Roncarolo MG (2013). Regulatory T cells: recommendations to simplify the nomenclature. Nat Immunol.

[145] Bluestone JA, Abbas AK (2003). Natural versus adaptive regulatory T cells. Nat Rev Immunol.

[146] Mackay LK, Kallies A (2017). Transcriptional regulation of tissue-resident lymphocytes. Trends Immunol.

[147] Jacobs JF, Nierkens S, Figdor CG, de Vries IJ, Adema GJ (2012). Regulatory T cells in melanoma: the final hurdle towards effective immunotherapy?. Lancet Oncol.

[148] Liyanage UK, Moore TT, Joo HG, Tanaka Y, Herrmann V, Doherty G, Drebin JA, Strasberg SM, Eberlein TJ, Goedegebuure PS (2002). Prevalence of regulatory T cells is increased in peripheral blood and tumor microenvironment of patients with pancreas or breast adenocarcinoma. J Immunol.

[149] Curiel TJ, Coukos G, Zou L, Alvarez X, Cheng P, Mottram P, Evdemon-Hogan M, Conejo-Garcia JR, Zhang L, Burow M (2004). Specific recruitment of regulatory T cells in ovarian carcinoma fosters immune privilege and predicts reduced survival. Nat Med.

[150] Shang B, Liu Y, Jiang SJ, Liu Y (2015). Prognostic value of tumor-infiltrating FoxP3+ regulatory T cells in cancers: a systematic review and meta-analysis. Sci Rep.

[151] Bates GJ, Fox SB, Han C, Leek RD, Garcia JF, Harris AL, Banham AH (2006). Quantification of regulatory T cells enables the identification of high-risk breast cancer patients and those at risk of late relapse. J Clinical Oncol.

[152] Tan W, Zhang W, Strasner A, Grivennikov S, Cheng JQ, Hoffman RM, Karin M (2011). Tumour-infiltrating regulatory T cells stimulate mammary cancer metastasis through RANKL-RANK signalling. Nature.

[153] Strauss L, Bergmann C, Whiteside TL (2009). Human circulating CD4+CD25highFoxp3+ regulatory T cells kill autologous CD8+ but not CD4+ responder cells by Fas-mediated apoptosis. J Immunol.

[154] Sojka DK, Huang YH, Fowell DJ (2008). Mechanisms of regulatory T-cell suppression - a diverse arsenal for a moving target. Immunology.

[155] Tanaka A, Sakaguchi S (2017). Regulatory T cells in cancer immunotherapy. Cell Res.

[156] Chen W, Jin W, Hardegen N, Lei KJ, Li L, Marinos N, McGrady G, Wahl SM (2003). Conversion of peripheral CD4+CD25- naive T cells to CD4+CD25+ regulatory T cells by TGF-beta induction of transcription factor Foxp3. J Exp Med.

[157] Facciabene A, Peng X, Hagemann IS, Balint K, Barchetti A, Wang LP, Gimotty PA, Gilks CB, Lal P, Zhang L (2011). Tumour hypoxia promotes tolerance and angiogenesis via CCL28 and T(reg) cells. Nature.

[158] Read S, Malmstrom V, Powrie F (2000). Cytotoxic T lymphocyte-associated antigen 4 plays an essential role in the function of CD25(+)CD4(+) regulatory cells that control intestinal inflammation. J Exp Med.

[159] Walker LS, Sansom DM (2015). Confusing signals: recent progress in CTLA-4 biology. Trends Immunol.

[160] Gabrilovich D (2004). Mechanisms and functional significance of tumour-induced dendritic-cell defects. Nat Rev Immunol.

[161] Lumeng CN, Saltiel AR (2011). Inflammatory links between obesity and metabolic disease. J Clin Invest.

[162] Mathis D (2013). Immunological goings-on in visceral adipose tissue. Cell Metab.

[163] Feuerer M, Herrero L, Cipolletta D, Naaz A, Wong J, Nayer A, Lee J, Goldfine AB, Benoist C, Shoelson S (2009). Lean, but not obese, fat is enriched for a unique population of regulatory T cells that affect metabolic parameters. Nat Med.

[164] Cipolletta D, Cohen P, Spiegelman BM, Benoist C, Mathis D (2015). Appearance and disappearance of the mRNA signature characteristic of Treg cells in visceral adipose tissue: age, diet, and PPARgamma effects. Proc Natl Acad Sci U S A.

[165] Cipolletta D, Feuerer M, Li A, Kamei N, Lee J, Shoelson SE, Benoist C, Mathis D (2012). PPAR-gamma is a major driver of the accumulation and phenotype of adipose tissue Treg cells. Nature.

[166] Hu ZQ, Zhao WH (2015). The IL-33/ST2 axis is specifically required for development of adipose tissue-resident regulatory T cells. Cell Mol Immunol.

[167] Fischer K, Hoffmann P, Voelkl S, Meidenbauer N, Ammer J, Edinger M, Gottfried E, Schwarz S, Rothe G, Hoves S (2007). Inhibitory effect of tumor cell-derived lactic acid on human T cells. Blood.

[168] Huber V, Camisaschi C, Berzi A, Ferro S, Lugini L, Triulzi T, Tuccitto A, Tagliabue E, Castelli C, Rivoltini L (2017). Cancer acidity: An ultimate frontier of tumor immune escape and a novel target of immunomodulation. Semin Cancer Biol.

[169] Lochner M, Berod L, Sparwasser T (2015). Fatty acid metabolism in the regulation of T cell function. Trends Immunol.

[170] Plitas G, Konopacki C, Wu K, Bos PD, Morrow M, Putintseva EV, Chudakov DM, Rudensky AY (2016). Regulatory T cells exhibit distinct features in human breast Cancer. Immunity.

[171] Angelin A, Gil-de-Gomez L, Dahiya S, Jiao J, Guo L, Levine MH, Wang Z, Quinn WJ, Kopinski PK, Wang L (2017). Foxp3 reprograms T cell metabolism to function in low-glucose, high-lactate environments. Cell Metab.

[172] Ohta A, Kini R, Ohta A, Subramanian M, Madasu M, Sitkovsky M (2012). The development and immunosuppressive functions of CD4(+) CD25(+) FoxP3(+) regulatory T cells are under influence of the adenosine-A2A adenosine receptor pathway. Front Immunol.

[173] Hosios AM, Hecht VC, Danai LV, Johnson MO, Rathmell JC, Steinhauser ML, Manalis SR, Vander Heiden MG (2016). Amino acids rather than glucose account for the majority of cell mass in proliferating mammalian cells. Dev Cell.

[174] Liberti MV, Locasale JW (2016). The Warburg effect: how does it benefit Cancer cells?. Trends Biochem Sci.

[175] Frauwirth KA, Riley JL, Harris MH, Parry RV, Rathmell JC, Plas DR, Elstrom RL, June CH, Thompson CB (2002). The CD28 signaling pathway regulates glucose metabolism. Immunity.

[176] Bantug GR, Galluzzi L, Kroemer G, Hess C. The spectrum of T cell metabolism in health and disease. Nat Rev Immunol. 2017. 10.1038/nri.2017.99 28944771

[177] Kinnaird A, Zhao S, Wellen KE, Michelakis ED (2016). Metabolic control of epigenetics in cancer. Nat Rev Cancer.

[178] van der Windt GJ, Everts B, Chang CH, Curtis JD, Freitas TC, Amiel E, Pearce EJ, Pearce EL (2012). Mitochondrial respiratory capacity is a critical regulator of CD8+ T cell memory development. Immunity.

[179] Doedens AL, Phan AT, Stradner MH, Fujimoto JK, Nguyen JV, Yang E, Johnson RS, Goldrath AW (2013). Hypoxia-inducible factors enhance the effector responses of CD8(+) T cells to persistent antigen. Nat Immunol.

[180] Phan AT, Doedens AL, Palazon A, Tyrakis PA, Cheung KP, Johnson RS, Goldrath AW (2016). Constitutive glycolytic metabolism supports CD8(+) T cell effector memory differentiation during viral infection. Immunity.

[181] Nishimura S, Manabe I, Nagasaki M, Eto K, Yamashita H, Ohsugi M, Otsu M, Hara K, Ueki K, Sugiura S (2009). CD8+ effector T cells contribute to macrophage recruitment and adipose tissue inflammation in obesity. Nat Med.

[182] Yang H, Youm YH, Vandanmagsar B, Ravussin A, Gimble JM, Greenway F, Stephens JM, Mynatt RL, Dixit VD (2010). Obesity increases the production of proinflammatory mediators from adipose tissue T cells and compromises TCR repertoire diversity: implications for systemic inflammation and insulin resistance. J Immunol.

[183] Winer S, Chan Y, Paltser G, Truong D, Tsui H, Bahrami J, Dorfman R, Wang Y, Zielenski J, Mastronardi F (2009). Normalization of obesity-associated insulin resistance through immunotherapy. Nat Med.

[184] Chi H (2012). Regulation and function of mTOR signalling in T cell fate decisions. Nat Rev Immunol.

[185] Geltink RIK, Kyle RL, Pearce EL (2018). Unraveling the complex interplay between T cell metabolism and function. Annu Rev Immunol.

[186] Ho PC, Bihuniak JD, Macintyre AN, Staron M, Liu X, Amezquita R, Tsui YC, Cui G, Micevic G, Perales JC (2015). Phosphoenolpyruvate is a metabolic checkpoint of anti-tumor T cell responses. Cell.

[187] Macintyre AN, Gerriets VA, Nichols AG, Michalek RD, Rudolph MC, Deoliveira D, Anderson SM, Abel ED, Chen BJ, Hale LP (2014). The glucose transporter Glut1 is selectively essential for CD4 T cell activation and effector function. Cell Metab.

[188] Wang R, Dillon CP, Shi LZ, Milasta S, Carter R, Finkelstein D, McCormick LL, Fitzgerald P, Chi H, Munger J (2011). The transcription factor Myc controls metabolic reprogramming upon T lymphocyte activation. Immunity.

[189] van der Windt GJ, O'Sullivan D, Everts B, Huang SC, Buck MD, Curtis JD, Chang CH, Smith AM, Ai T, Faubert B (2013). CD8 memory T cells have a bioenergetic advantage that underlies their rapid recall ability. Proc Natl Acad Sci U S A.

[190] Chang CH, Curtis JD, Maggi LB, Faubert B, Villarino AV, O'Sullivan D, Huang SC, van der Windt GJ, Blagih J, Qiu J (2013). Posttranscriptional control of T cell effector function by aerobic glycolysis. Cell.

[191] Sena LA, Li S, Jairaman A, Prakriya M, Ezponda T, Hildeman DA, Wang CR, Schumacker PT, Licht JD, Perlman H (2013). Mitochondria are required for antigen-specific T cell activation through reactive oxygen species signaling. Immunity.

[192] Nakagawa Y, Negishi Y, Shimizu M, Takahashi M, Ichikawa M, Takahashi H (2015). Effects of extracellular pH and hypoxia on the function and development of antigen-specific cytotoxic T lymphocytes. Immunol Lett.

[193] Patsoukis N, Bardhan K, Chatterjee P, Sari D, Liu B, Bell LN, Karoly ED, Freeman GJ, Petkova V, Seth P (2015). PD-1 alters T-cell metabolic reprogramming by inhibiting glycolysis and promoting lipolysis and fatty acid oxidation. Nat Commun.

[194] Cekic C, Linden J (2014). Adenosine A2A receptors intrinsically regulate CD8+ T cells in the tumor microenvironment. Cancer Res.

[195] Hiller JG, Cole SW, Crone EM, Byrne DJ, Shackleford DM, Pang JB, Henderson MA, Nightingale SS, Ho KM, Myles PS (2020). Preoperative beta-blockade with propranolol reduces biomarkers of metastasis in breast Cancer: a phase II randomized trial. Clinical Cancer Res.

[196] Daher C, Vimeux L, Stoeva R, Peranzoni E, Bismuth G, Wieduwild E, Lucas B, Donnadieu E, Bercovici N, Trautmann A (2019). Blockade of beta-adrenergic receptors improves CD8(+) T-cell priming and Cancer vaccine efficacy. Cancer immunology research.

[197] Konger RL, Derr-Yellin E, Ermatov N, Ren L, Sahu RP (2019). The PPARgamma Agonist Rosiglitazone Suppresses Syngeneic Mouse SCC (Squamous Cell Carcinoma) Tumor Growth through an Immune-Mediated Mechanism. Molecules.

[198] Bunt SK, Mohr AM, Bailey JM, Grandgenett PM, Hollingsworth MA (2013). Rosiglitazone and gemcitabine in combination reduces immune suppression and modulates T cell populations in pancreatic cancer. Cancer immunology, immunotherapy.

[199] Ladanyi A, Mukherjee A, Kenny HA, Johnson A, Mitra AK, Sundaresan S, Nieman KM, Pascual G, Benitah SA, Montag A, et al. Adipocyte-induced CD36 expression drives ovarian cancer progression and metastasis. Oncogene. 2018. 10.1038/s41388-017-0093-z PMC5920730 29398710

[200] Wang H, Franco F, Tsui YC, Xie X, Trefny MP, Zappasodi R, Mohmood SR, Fernandez-Garcia J, Tsai CH, Schulze I (2020). CD36-mediated metabolic adaptation supports regulatory T cell survival and function in tumors. Nat Immunol.

[201] Polanski R, Hodgkinson CL, Fusi A, Nonaka D, Priest L, Kelly P, Trapani F, Bishop PW, White A, Critchlow SE (2014). Activity of the monocarboxylate transporter 1 inhibitor AZD3965 in small cell lung cancer. Clin Cancer Res.

[202] Xie H, Hanai J, Ren JG, Kats L, Burgess K, Bhargava P, Signoretti S, Billiard J, Duffy KJ, Grant A (2014). Targeting lactate dehydrogenase--a inhibits tumorigenesis and tumor progression in mouse models of lung cancer and impacts tumor-initiating cells. Cell Metab.

[203] Boison D, Yegutkin GG (2019). Adenosine metabolism: emerging concepts for Cancer therapy. Cancer Cell.

[204] Ribas A, Wolchok JD (2018). Cancer immunotherapy using checkpoint blockade. Science.

[205] Afzal MZ, Mercado RR, Shirai K (2018). Efficacy of metformin in combination with immune checkpoint inhibitors (anti-PD-1/anti-CTLA-4) in metastatic malignant melanoma. J Immunother Cancer.

[206] Eikawa S, Nishida M, Mizukami S, Yamazaki C, Nakayama E, Udono H (2015). Immune-mediated antitumor effect by type 2 diabetes drug, metformin. Proc Natl Acad Sci U S A.

[207] Reggiani F, Labanca V, Mancuso P, Rabascio C, Talarico G, Orecchioni S, Manconi A, Bertolini F (2017). Adipose progenitor cell secretion of GM-CSF and MMP9 promotes a stromal and immunological microenvironment that supports breast Cancer progression. Cancer Res.

[208] Incio J, Ligibel JA, McManus DT, Suboj P, Jung K, Kawaguchi K, Pinter M, Babykutty S, Chin SM, Vardam TD (2018). Obesity promotes resistance to anti-VEGF therapy in breast cancer by up-regulating IL-6 and potentially FGF-2. Sci Transl Med.

[209] Kolb R, Phan L, Borcherding N, Liu Y, Yuan F, Janowski AM, Xie Q, Markan KR, Li W, Potthoff MJ (2016). Obesity-associated NLRC4 inflammasome activation drives breast cancer progression. Nat Commun.

[210] Foretz M, Guigas B, Bertrand L, Pollak M, Viollet B (2014). Metformin: from mechanisms of action to therapies. Cell Metab.

[211] Cha JH, Yang WH, Xia W, Wei Y, Chan LC, Lim SO, Li CW, Kim T, Chang SS, Lee HH (2018). Metformin promotes antitumor immunity via endoplasmic-reticulum-associated degradation of PD-L1. Mol Cell.

[212] Yang H, Yamazaki T, Pietrocola F, Zhou H, Zitvogel L, Ma Y, Kroemer G (2015). STAT3 inhibition enhances the therapeutic efficacy of immunogenic chemotherapy by stimulating type 1 interferon production by Cancer cells. Cancer Res.

